# Immune checkpoint protein VSIG4 as a biomarker of aging in murine adipose tissue

**DOI:** 10.1111/acel.13219

**Published:** 2020-08-28

**Authors:** Brandon M. Hall, Anatoli S. Gleiberman, Evguenia Strom, Peter A. Krasnov, David Frescas, Slavoljub Vujcic, Olga V. Leontieva, Marina P. Antoch, Valeria Kogan, Igor E. Koman, Yi Zhu, Tamara Tchkonia, James L. Kirkland, Olga B. Chernova, Andrei V. Gudkov

**Affiliations:** ^1^ Everon Biosciences Inc Buffalo NY USA; ^2^ Department of Pharmacology and Therapeutics Roswell Park Comprehensive Cancer Center Buffalo NY USA; ^3^ Institute for Translational Research Ariel University Ariel Israel; ^4^ Robert and Arlene Kogod Center on Aging Mayo Clinic Rochester MN USA; ^5^ Department of Cell Stress Biology Roswell Park Comprehensive Cancer Center Buffalo NY USA; ^6^ Genome Protection Inc Buffalo NY USA

**Keywords:** adipose tissue, aging, frailty index, immune checkpoint, inflammation, macrophage, mouse, VSIG4

## Abstract

Adipose tissue is recognized as a major source of systemic inflammation with age, driving age‐related tissue dysfunction and pathogenesis. Macrophages (Mφ) are central to these changes yet adipose tissue Mφ (ATMs) from aged mice remain poorly characterized. To identify biomarkers underlying changes in aged adipose tissue, we performed an unbiased RNA‐seq analysis of ATMs from young (8‐week‐old) and healthy aged (80‐week‐old) mice. One of the genes identified, V‐set immunoglobulin‐domain‐containing 4 (VSIG4/CRIg), encodes a Mφ‐associated complement receptor and B7 family‐related immune checkpoint protein. Here, we demonstrate that *Vsig4* expression is highly upregulated with age in perigonadal white adipose tissue (gWAT) in two mouse strains (inbred C57BL/6J and outbred NIH Swiss) independent of gender. The accumulation of VSIG4 was mainly attributed to a fourfold increase in the proportion of VSIG4^+^ ATMs (13%–52%). In a longitudinal study, VSIG4 expression in gWAT showed a strong correlation with age within a cohort of male and female mice and correlated strongly with physiological frailty index (PFI, a multi‐parameter assessment of health) in male mice. Our results indicate that VSIG4 is a novel biomarker of aged murine ATMs. VSIG4 expression was also found to be elevated in other aging tissues (e.g., thymus) and was strongly induced in tumor‐adjacent stroma in cases of spontaneous and xenograft lung cancer models. VSIG4 expression was recently associated with cancer and several inflammatory diseases with diagnostic and prognostic potential in both mice and humans. Further investigation is required to determine whether VSIG4‐positive Mφ contribute to immunosenescence and/or systemic age‐related deficits.

## Graphical abstract

Adipocytes were removed from gonadal fat (gWAT) of old and young mice, and remaining cells were fractionated into cells capable (e.g., Mφ) and incapable of phagocyting magnetic iron particles. RNA was sequenced and analyzed to identify aging‐associated macrophage‐specific transcripts. Identified candidates were validated, and VSIG4 was confirmed a macrophageal biomarker correlating with chronological and biological age.

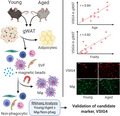

## 1 INTRODUCTION

Mφ are sentinels of the immune system, detecting and responding to various stressors (e.g., infection, tissue damage, cell transformation) in order to maintain tissue homeostasis (Wynn, Chawla, & Pollard, 2013). Mφ play pivotal roles in clearing pathogens and cellular debris, modulating both innate and adaptive branches of immunity, and orchestrating tissue remodeling, inflammatory response, and subsequent resolution. Aging is associated with a general decline of proper immune function. Age‐related changes in Mφ are associated with delayed wound healing, cancer progression, and the development of age‐associated inflammatory diseases (e.g., atherosclerosis, rheumatoid arthritis, and peripheral neuropathy; Becker et al., 2017; Mahbub, Deburghgraeve, & Kovacs, 2012; Oishi & Manabe, 2016; Rowe et al., 2016; Thevaranjan et al., 2017; Yuan et al., 2018).

Adipose tissue is regarded as the largest contributor to systemic inflammation with age (Palmer & Kirkland, 2016; Starr et al., 2009; Stout, Justice, Nicklas, & Kirkland, 2017; Wu et al., 2007). Aged adipose tissue is characterized by altered phenotypes and cellular responses, marked by a shift in several cell types toward a pro‐inflammatory phenotype, including adipose tissue macrophages (ATMs) (van Beek, Van den Bossche, Mastroberardino, de Winther, & Leenen, 2019; Blomberg, Diaz, Romero, Vasquez, & Frasca, 2016; Camell et al., 2019; Garg, Delaney, Shi, & Yung, 2014; Kalathookunnel Antony, Lian, & Wu, 2018; Lumeng et al., 2011). Adipose tissue dysfunction and resultant pro‐inflammatory cytokine secretion (e.g., IL‐6, IL‐1β, TNF‐α) are associated with systemic dysregulation of tissue homeostasis and an increase in organismal frailty (Stout et al., 2017). In the context of diet‐induced obesity, adipose tissue dysfunction results from the infiltration and proliferation of pro‐inflammatory Mφ, the main source of secreted cytokines (Lumeng, DeYoung, Bodzin, & Saltiel, 2007). Importantly, extensive Mφ infiltration has not been observed during aging, and while mechanisms of age‐induced adipose tissue dysfunction are poorly understood, recent research has drawn a distinction from obesity‐related mechanisms (Bapat, Suh, et al., 2015; Garg et al., 2014; Kalathookunnel Antony et al., 2018; Lumeng et al., 2011; Tchkonia et al., 2010). Given their central role in inflammation and adipose tissue homeostasis, characterization of ATMs is essential to understanding processes underlying adipose tissue dysfunction with age.

Since aged ATMs were recently shown to possess fundamental markers of aging associated with frailty (e.g., *Il6*, *p16^Ink4a^*, senescence‐associated β‐galactosidase activity) (Hall et al., 2016, 2017; Lumeng et al., 2011), we performed whole transcriptome analysis as an unbiased approach to identify age‐related differential gene expression in perigonadal ATMs. Genes exhibiting increased Mφ‐associated expression with age were chosen for confirmation as candidate biomarkers of aged adipose tissue and were subsequently validated for their differential gene expression in mice of two genetic backgrounds (C57BL/6J and NIH Swiss). One of the genes identified, VSIG4 (CRIg/Z39Ig), was of interest as a Mφ‐associated protein with roles spanning both innate and adaptive immunity. As a tissue‐resident Mφ marker, VSIG4 is a multifunctional cell surface protein with potent immunosuppressive and anti‐inflammatory roles [as recently reviewed (Small, Al‐Baghdadi, Quach, Hii, & Ferrante, 2016)]. Notably, induction of VSIG4 expression is associated with several inflammatory diseases and cancers in both mice and humans. VSIG4 functions as an immune checkpoint regulator, suppressing T‐lymphocyte function and promoting cancer development and progression (Bianchi‐Frias et al., 2019; Liao et al., 2014). In this study, we demonstrate that increased VSIG4 expression by ATMs correlates with age and physiological frailty in mice, suggesting that accumulation of VSIG4 may reflect relevant phenotypic changes that underlie age‐related pathologies. Thus, we present VSIG4 as a novel biomarker of aged murine ATMs. Further studies are required to elucidate whether elevated levels of VSIG4 in adipose and other tissues promote age‐related deficits, such as immunosenescence, dysregulation of tissue homeostasis, and/or tumor establishment.

## 2 RESULTS

### 2.1 Identification of *Vsig4* as a biomarker of aged ATMs

**FIGURE 1:**
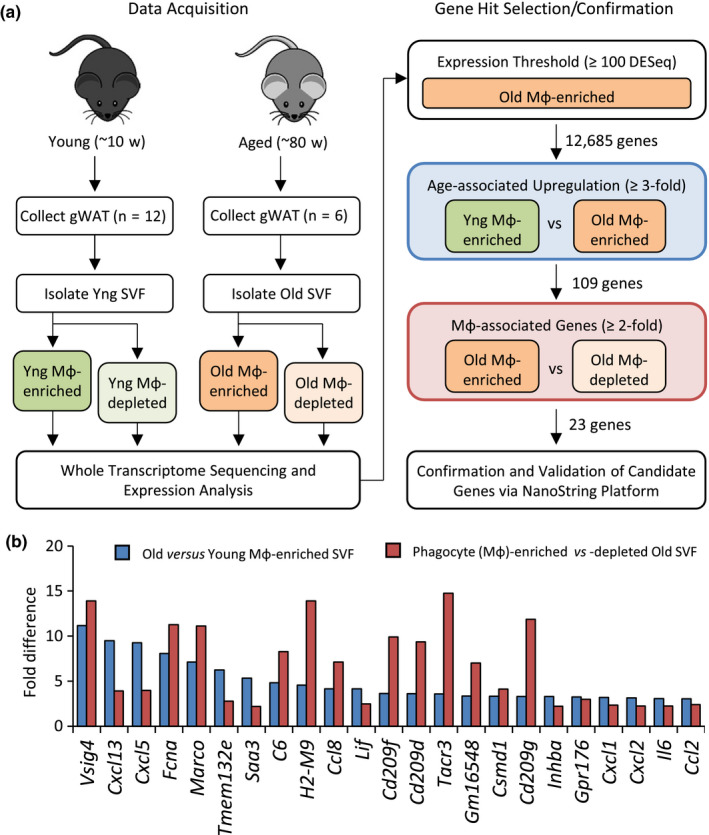
Schematic of data acquisition and identification of genes upregulated in Mφ from old mice. (a) The stromal vascular fraction (SVF) was isolated *via* enzymatic digestion of perigonadal white adipose tissue (gWAT) pooled from young (Yng; *n* = 12) or chronologically aged (Old; *n* = 6) mice. Mφ were then segregated into Mφ‐enriched and resultant Mφ‐depleted fractions (see Section 4). Total RNA was subsequently extracted and analyzed *via* whole transcriptome sequencing. Following data processing and quantitation of expression, genes were filtered using several criteria, including (i) expression level, (ii) upregulation in aged Mφ, (iii) and Mφ‐associated gene expression (i.e., genes enriched in Mφ‐enriched fraction). Confirmation of age‐dependent differential gene expression in adipose tissue Mφ was performed *via* NanoString analysis, using the same RNA extracts submitted for RNA‐seq analysis (Table S2). (b) Differential gene expression of analyzed RNA‐seq data, with genes ranked by the extent of upregulation in the ATM‐enriched SVF between age groups (blue bars). For each gene, the extent of enrichment between the ATM‐enriched and ATM‐depleted SVF from aged mice is depicted (red bars)

To identify Mφ‐specific biomarkers that accumulate with age in mice, we performed unbiased RNA sequencing analysis on adipose tissue Mφ (ATMs) enriched from the stromal vascular fraction (SVF) of pooled perigonadal white adipose tissues (gWAT) of young (8‐week‐old) and naturally aged (80‐week‐old) C57BL/6J mice (Figure 1a). Mφ were enriched using a novel method based on the ability of Mφ to phagocytize superparamagnetic nanoparticles, enabling subsequent enrichment away from non‐phagocytic cells *via* magnetic‐activated cell sorting. Gene expression analysis of RNA‐seq data revealed enrichment of classical Mφ‐specific markers in the phagocyte‐enriched versus phagocyte‐depleted populations of SVF (Table S1). In contrast, analysis of several internal reference genes (*B2m*, *Sap130*, *Sdha*, *Tbp*, *Tubb5*) was similar across analyzed samples (Table S1).

Mφ‐associated genes exhibiting age‐related upregulation were then selected based on the following criteria: (a) expression level (≥100 normalized counts in the Mφ‐enriched SVF from aged mice), (b) age‐associated upregulation (≥3‐fold difference in Mφ‐enriched SVFs between young and aged mice), and (c) Mφ‐associated gene expression (≥2‐fold enrichment in the Mφ‐enriched versus Mφ‐depleted SVFs from aged mice) (Figure 1a). A total of 23 candidate genes were identified (Figure 1b), 20 of which were selected for confirmation studies. To evaluate the list of candidate genes, we confirmed age‐dependent differential gene expression using a custom‐built NanoString Technologies nCounter^®^ codeset platform. Analysis of the Mφ‐enriched SVF samples used for RNA‐seq confirmed that 17 genes (85%) exhibited a ≥3‐fold age‐dependent increase in expression (Table S2). Differential gene expression for the remaining 3 genes (*Csmd1*, *Tacr3*, and *Tmem132e*) could not be accurately assessed due to low‐level expression. The majority of confirmed genes encoded secreted cytokines and chemokines previously associated with inflammation and/or aging (*Ccl2*,* Ccl8*,* Cxcl1*,* Cxcl2*,* Cxcl5*,* Cxcl13*,* Il6*,* Inhba*,* Lif*,* Saa3*) (Song et al., 2012; Yousefzadeh, Schafer, et al., 2018; Hudgins et al., 2018; Greene & Loeser, 2015; Loria et al., 1998; Xu, Palmer, et al., 2015; Knight, 2001; Den Hartigh et al., 2014artigh et al., 2014), although not all of these genes have been linked to age‐related processes in adipose tissue and/or Mφ. In addition, we confirmed an age‐related increase of Mφ‐associated genes encoding surface receptors with roles in phagocytosis, including collagen‐like scavenger receptor Marco, several C‐type lectin receptors (Cd209d, Cd209f, and Cd209g), and complement receptor and immune checkpoint protein, *Vsig4*.

**FIGURE 2:**
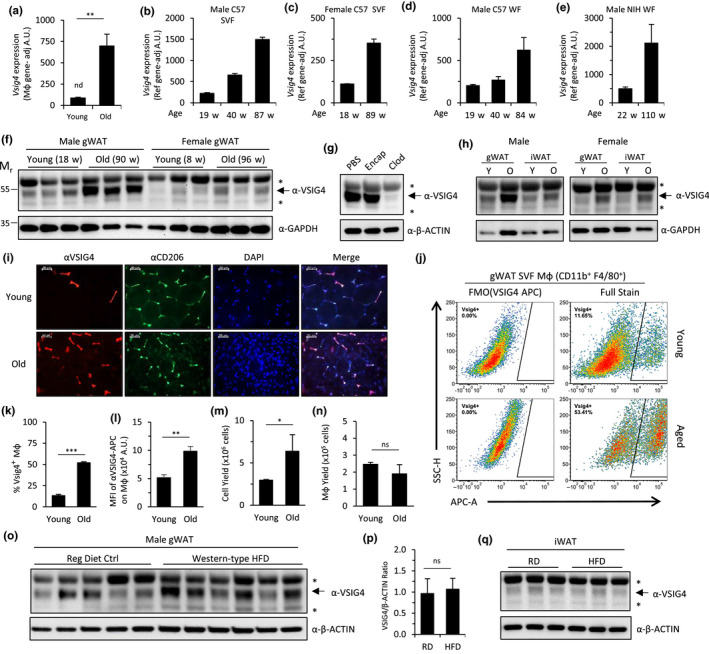
Age‐related increase of *Vsig4* gene expression in murine adipose tissue. (a–e) NanoString gene expression analysis of *Vsig4* in gWAT samples from young and old mice. (a) *Vsig4* mRNA expression analysis of Mφ‐enriched SVF from several independent pools of 3–12 mice each, either young (8‐week‐old; *n* = 2 pools) or aged (78‐ to 88‐week‐old; *n* = 3 pools), adjusted to gene expression of three pan‐Mφ genes *Adgre1*, *Mrc1*, and *Csf1r*. Data presented as mean ± *SD*; nd = not detectable. **p < .01, compared to young mice. *Vsig4* mRNA expression analysis of total SVF of gWAT from different‐aged cohorts of male (b) or female (c) mice, and of intact gWAT from male C57BL/6J (d) and NIH Swiss (e) mice, adjusted to gene expression of five internal reference genes *B2m*, *Sap130*, *Sdha*, *Tbp*, and *Tubb5*. Data presented as a single measurement of pooled samples (*n* = 3–8 mice/group) ± error propagation from *SD* of normalized internal reference genes. (f–h) Immunoblot analysis of age‐dependent VSIG4 expression from intact adipose tissues of (f) gWAT from three individual male and female C57BL/6J mice; (g) gWAT from old male C57BL/6J mice (102‐week‐old) 1 week after a single administration of PBS, Encapsome (Encap; liposomal PBS), or Mφ depletion reagent Clodrosome (Clod; liposomal clodronate); (h) pooled samples (*n* = 3 mice) of gWAT and inguinal white adipose tissue (iWAT) collected from young (Y; 8‐ to 18‐week‐old) and old (O; 90‐ to 96‐week‐old) C57BL/6J mice. GAPDH or β‐actin were used as immunoblot loading controls, as indicated. *Non‐specific α‐VSIG4 immunoreactive bands, as indicated in panel (g). (i) Representative fluorescence microphotographs of intact gWAT from young (14‐week‐old) and old (84‐week‐old) mice depicting immunofluorescent staining of Mφ‐related markers VSIG4 (red) and CD206 (green), and a merged overlay with DAPI nuclear counterstain (blue). Scale bar = 50‐μm. (j–n) Representative experiment of flow cytometric analysis of single‐cell suspensions of the SVF isolated from three individual young (19‐week‐old) and old (120‐week‐old) male C57BL/6J mice. (j) Pseudocolor plots from flow cytometric analysis of VSIG4 expression in live‐stained, viable ATMs (CD45^+^ CD11b^+^ F4/80^+^). All gates were defined using age‐matched fluorescence minus one (FMO) controls (see gating scheme in Figure S6A,B). Quantitation of (k) the proportion of VSIG4^+^ Mφ and (l) the median fluorescent intensity (MFI) of αVSIG4‐APC stained Mφ minus MFI from negative control staining, that is, FMO(VSIG4‐APC). (m) SVF total cell yield per mouse. (n) Total Mφ yield from gWAT per mouse, estimated from the proportion of SVF ATMs (CD11b^+^ F4/80^+^). Data presented as mean ± *SD* (*n* = 3 mice/group). ns, not significant (*p* > .05), *p < .05, **p < .01, ***p < .001, compared to young mice. Results are representative of 3 independent experiments with 3‐6 mice per group (processed as individual or pooled samples). (o–q) Immunoblot analysis of VSIG4 in gWAT (o) and iWAT (q) from individual male C57BL/6J mice after 7 months of control diet (regular diet ctrl; RD) or a “Western”‐type high‐calorie diet (Western‐type HFD; HFD) feeding *ad libitum*. (p) Densitometric analysis of VSIG4 band intensity in gWAT samples (normalized to β‐actin). Data presented as mean ± *SD* (*n* = 5‐6 mice/group). ns, not significant (*p* > 0.05). β‐actin was used as a loading control. *Non‐specific α‐VSIG4 immunoreactive bands, as indicated in panel (g)

Analysis of several independent pools of Mφ‐enriched SVF revealed the strongest and most consistently upregulated genes in aged male mice to be tissue‐resident Mφ marker *Vsig4*, cytokine *Saa3*, and chemokines *Ccl8* (*Mcp2*), *Cxcl5*, and *Cxcl13* (Figure S1A). Of these genes, *Vsig4* was of significant interest as a gene not previously associated with aging, encoding a multifaceted cell surface receptor with roles in both innate and adaptive immunity (Small et al., 2016). *Vsig4* was undetectable in ATM‐enriched SVF from young mice but showed a substantial signal in aged mice (8.0‐fold increase above background signal; *p* = .008; Figure 2a).

### 2.2 Age‐related upregulation of Vsig4 in adipose tissue is independent of gender and genetic background

An ideal biomarker is one that is reliably associated with a specific condition and can be assessed following minimal tissue processing. Therefore, we assessed the expression of *Vsig4* and other candidate genes in total SVF (without Mφ enrichment) and whole adipose tissue to determine whether these samples were suitable for the detection of differential expression between young and old mice *via* the NanoString platform. Further, we examined whether age‐dependent increases were influenced by gender and/or genetic background. Perigonadal fat from young male C57BL/6J mice exhibited elevated levels of *Vsig4* expression compared to young female mice (2.0‐fold difference) (Figure 2b,c). Aged male mice showed a greater increase in *Vsig4* expression compared to female littermates (6.7‐fold and 3.2‐fold increase, respectively). Analysis of intact gWAT from both inbred C57BL/6J and outbred NIH Swiss mice also demonstrated an age‐dependent increase in *Vsig4* expression (3.1‐fold and 4.3‐fold, respectively; Figure 2d,e). Notably, as the only gene to show a ≥3‐fold increase across all samples, *Vsig4* was validated as our strongest biomarker candidate (Figure S1B). Gene expression of other Mφ‐specific markers (*Adgre1*, *Csf1r*, *Mrc1*) was analyzed in parallel, revealing minor changes relative to *Vsig4*; in the SVF and intact gWAT from C57BL/6J mice, a ≤1.5‐fold age‐related increase was observed in classical Mφ markers, whereas gWAT from NIH Swiss mice exhibited up to a twofold decrease (Figure S2A–D). These data indicate that, unlike other Mφ‐specific markers, *Vsig4* is strongly upregulated with age. In summary, our efforts to validate age‐related gene expression in gWAT revealed a strong, consistent increase of *Vsig4* expression in two strains of chronologically aged mice, C57BL/6J and NIH Swiss, independent of gender.

### 2.3 Age‐dependent increase in the proportion of VSIG4‐positive ATMs in gWAT

We next evaluated age‐related changes in VSIG4 protein expression in gWAT *via* immunoblot analysis. We found that gWAT from males expressed more VSIG4 compared to females and that both genders displayed an age‐dependent increase of VSIG4 (Figure 2f), consistent with *Vsig4* mRNA expression. VSIG4 expression was relatively homogenous through the length of the gWAT pad in young and old male mice (Figure S3A–C). To evaluate Mφ‐specific immunoreactivity of the anti‐VSIG4 antibody against lysates of gWAT, aged mice were treated with liposomal clodronate, a Mφ‐specific depletion reagent. gWAT from clodronate‐treated mice exhibited a loss of immunofluorescent staining of Mφ markers VSIG4 and CD301a in whole fat (Figure S4). Concomitantly, we observed a dramatic reduction in the age‐associated anti‐VSIG4 immunoreactive band *via* immunoblot (Figure 2g). Other bands of relatively higher and lower molecular weight were unchanged, indicating their non‐specific immunoreactivity. These data indicate that VSIG4 expression of ATMs was elevated in aged mice consistent with *Vsig4* transcript expression, with male mice exhibiting substantially higher levels in gWAT than female mice from the same cohort.

To determine whether the increased expression of VSIG4 in adipose tissue with age was site‐specific, we characterized several adipose tissue depots *via* immunoblot. VSIG4 was expressed in both gWAT and iWAT from young mice and was substantially increased with age in both male and female C57BL/6J and male NIH Swiss mice (Figure 2h; Figure S5A). However, VSIG4 expression in perirenal white adipose tissue (rWAT) was highly variable with no apparent age‐related trend, and in brown adipose tissue (BAT) isolated from the interscapular depot, little to no VSIG4 expression was observed (Figure S5A). Expression analysis was also expanded to a panel of several tissues, revealing that, while most tissues either lack substantial levels of VSIG4 expression or do not show an appreciable increase with age, the heart, thymus, and peritoneal cavity exhibit a strong accumulation of VSIG4 in aged mice (Figure S5B–H). In the thymus, immunofluorescence analysis revealed an extensive increase of VSIG4‐positive Mφ in the remnants of the cortex following thymic involution (Figure S5H). Overall, our analysis reveals a substantial tissue‐specific accumulation of VSIG4 expression with age.

We next characterized age‐related expression of VSIG4 in gWAT *via* immunofluorescent staining. In both young and aged mice, VSIG4 staining co‐localized with staining for tissue‐resident Mφ marker CD206 (Figure 2i), demonstrating Mφ‐specific expression of VSIG4 in aged adipose tissue. In the different conditions and tissues we analyzed (including others not presented in this manuscript), VSIG4‐positive cells were always a subpopulation of Mφ (defined by pan‐Mφ markers, e.g., F4/80 and CD11b) that express other tissue‐resident Mφ markers (CD206^+^, CD209a^+^, and/or CD301a^+^). The percentage of CD206^+^ ATMs expressing VSIG4 was increased from 17.6 +/− 4.7% VSIG4‐positive ATMs in young mice to 61.1 +/− 4.4% in old mice (*p* <.001), with most ATMs expressing VSIG4. To quantify the proportion of VSIG4^+^ Mφ and relative VSIG4 expression per cell, the SVF isolated from gWAT in young and old male C57BL/6J mice was analyzed *via* flow cytometry (see Figure S6A,B for gating scheme). Notably, the anti‐VSIG4 antibody used for flow cytometry showed strict co‐localization with the different VSIG4 antibody used for immunofluorescent staining (Figure S7). The percentage of VSIG4^+^ ATMs (CD11b^+^F4/80^+^) in aged gWAT was elevated 3.9‐fold (*p* < .001) compared to young mice, increasing from 13% to 52% (Figure 2j,k). In addition, we observed a 1.9‐fold increase in the median fluorescent intensity of VSIG4^+^ ATMs with age (Figure 2l). Despite higher yields of SVF cells isolated from aged gWAT, the total number of ATMs was found to be similar between young and aged mice (Figure 2m,n). Together, these data indicate that the increased abundance of VSIG4 in aged gWAT is attributed to both an increased proportion of tissue‐resident Mφ expressing VSIG4 and an age‐dependent increase in VSIG4 expression within the VSIG4^+^ Mφ population.

### 2.4 VSIG4 protein levels elevated with age but not diet‐induced obesity

VSIG4 expression is associated with several inflammatory diseases that are linked to aging. Both aging and diet‐induced obesity (DIO) provoke inflammation in adipose tissue, albeit with differing etiology (Garg et al., 2014; Lumeng et al., 2011). We examined whether VSIG4 expression was upregulated in response to changes in adipose tissue elicited by obesity. Expression of VSIG4 was analyzed in gWAT and iWAT from obese, Western‐type high‐fat diet‐fed mice exhibiting increased intraabdominal fat and signs of impaired glucose tolerance (Figure S8A–E). Immunoblot analysis revealed no differences in VSIG4 expression in gWAT or iWAT from DIO mice compared to normal diet‐fed, age‐matched controls (Figure 2o–q). Analysis of gWAT tissue *via* immunofluorescent staining indicates that, unlike aging, DIO is associated with an extensive infiltration of Mφ into adipose tissues, consistent with literature data (Bapat, Suh, et al., 2015; Li et al., 2017; Figure S8F). However, the proportion of VSIG4^+^ Mφ was decreased in DIO mice, suggesting that infiltrating Mφ are largely negative for VSIG4 expression. Robust *Vsig4* upregulation was associated with aging but not DIO, reflecting intrinsic differences between these two conditions.

### 2.5 VSIG4 expression is associated with cancer

**FIGURE 3:**
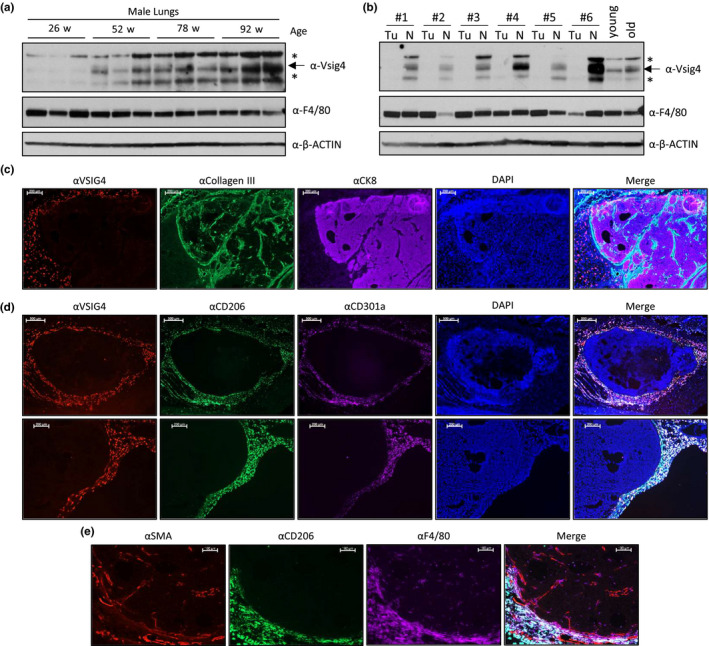
VSIG4 expression induced by aging and cancer. Immunoblot analysis of (a) normal lung tissue from individual NIH Swiss mice of different ages (26–96 weeks old) and (b) tumors (Tu) and normal tumor‐adjacent lung tissue (N) from individual aged mice bearing spontaneous lung cancer. (c–e) Representative fluorescence microphotographs of A549 xenografts grown subcutaneously in severe immunocompromised (SCID) mice, depicting immunofluorescent staining of (c) VSIG4 (red), stromal marker collagen III (green), epithelial marker cytokeratin 8 (CK8) for labeling of A549 cells (scale bar = 200‐μm); (d) VSIG4 (red) and tissue‐resident Mφ markers CD206 (green) and CD301a (purple) (scale bar = 500‐μm [top] and 200‐μm [bottom]); and (e) stromal marker smooth muscle actin (SMA; red), CD206 (green), and pan‐Mφ marker F4/80 (purple). DAPI nuclear counterstain (blue) and merged overlay of colored channels are depicted

VSIG4, an immune checkpoint protein, is thought to enable the cancer progression through suppression of T‐lymphocyte activation (Bianchi‐Frias et al., 2019; Liao et al., 2014). NIH Swiss mice exhibit a relatively high incidence of spontaneous lung cancer with age. Unlike C56BL/6J mice, NIH Swiss mice exhibited an age‐dependent increase in VSIG4 expression in tissue from normal lungs, that is, lacking gross pathology (Figure 3a). Immunoblot analysis of VSIG4 expression in lungs bearing cancer lesion(s) revealed that expression was undetectable within the primary tumors (Figure 3b). However, within the normal, tumor‐adjacent tissue, VSIG4 was highly expressed. Similar observations were found in a xenograft model of A549 lung adenocarcinoma in immunocompromised mice, wherein immunofluorescent staining revealed an abundant population of tissue‐resident Mφ (CD206‐ and/or CD301a‐positive) expressing VSIG4 within the normal stroma on the tumor periphery (Figure 3c,d). In contrast, anti‐VSIG4 staining was absent within the tumor microenvironment and was less frequent among tissue‐resident Mφ in the skin further away from the tumor margin (Figure 3d). The presence of Mφ infiltrates within the tumor was confirmed by F4/80+ CD206‐ staining (Figure 3e). These findings indicate that VSIG4 can be induced by tissue‐resident Mφ in the immediate vicinity of cancer lesions.

### 2.6 VSIG4 expression in gWAT correlates with healthspan

**FIGURE 4:**
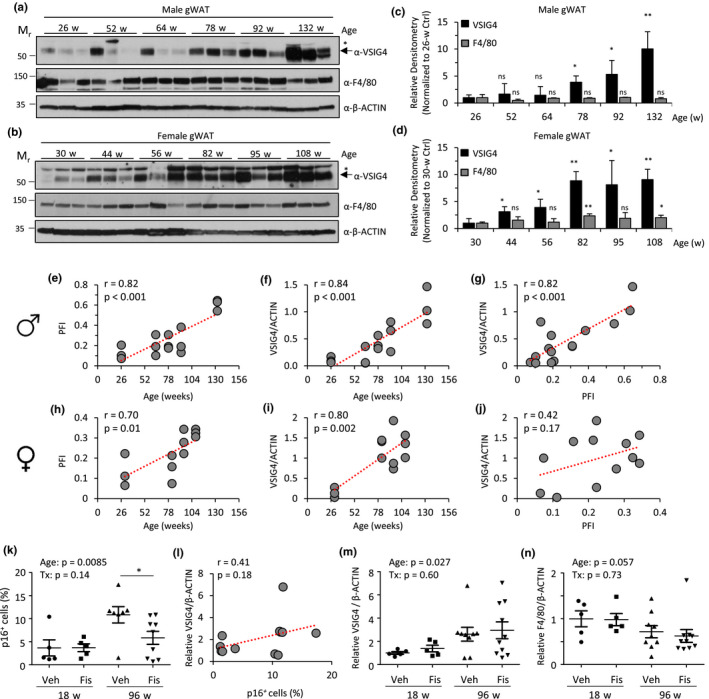
Correlation of VSIG4 expression in gWAT with chronological age and health throughout life. (a–j) A longitudinal study of a single cohort of chronologically aged male and female NIH Swiss littermates, where physiological frailty index (PFI; a multi‐parameter assessment of health) was analyzed throughout the life span of the mice. At indicated ages, a final PFI was performed and gWAT was collected. Immunoblot analysis of VSIG4 and Mφ marker F4/80 in gWAT from individual male (a) and female (b) mice (*n* = 3). β‐actin was used as a loading control. *Non‐specific α‐VSIG4 immunoreactive bands. Densitometric analysis of immunoblot images for male (c) and female (d) mice, evaluating the expression level of VSIG4 and F4/80 with respect to β‐actin for each mouse. Data represent mean ± *SD* of values normalized to the youngest group for each gender. ns, not significant (*p* > .05), *p < .05, **p < .01, compared to young mice (26‐ or 30‐week‐old mice for males and females, respectively). (e–j) Correlation of VSIG4 immunoblot densitometric analysis (normalized to β‐actin), chronological age and PFI in male (e–g) and female (h–j) mice: (e, h) age versus PFI, (f, i) VSIG4 expression versus age, and (g, j) VSIG4 expression versus PFI. Pearson correlation coefficient (*r*) indicated in top left corner of graph. Exact *p*‐values are indicated. (k–n) Analysis of gWAT samples from female INK‐ATTAC mice collected 5 days after two consecutive daily treatments with either vehicle or fisetin (100 mg/kg per os). (k) Quantitation of proportion of p16*^Ink4a^*‐expressing SVF cells, as assessed by CyToF analysis of anti‐Flag immunoreactivity (INK‐ATTAC mice harbor transgenic construct for p16*^Ink4a^* promoter‐driven Flag‐tagged FKBP‐caspase 8 fusion protein). (l) Correlation of VSIG4 immunoblot densitometric analysis (normalized to β‐actin and expressed as the relative difference compared to young vehicle‐treated mice) and the proportion of p16*^Ink4a^*‐expressing cells. Densitometric analysis of VSIG4 (m) or F4/80 (n) immunoblots (normalized to β‐actin) of gWAT for individual samples, depicted as the relative expression compared to young vehicle‐treated mice. A two‐way ANOVA (age and treatment effects indicated as exact *p*‐values) with Bonferroni *post hoc* test for comparison between treatment groups (*p < .05; otherwise not significant, *p* > .05)

VSIG4 expression was found to be highly upregulated in aged mice; however, it is unclear why VSIG4 expression changes and whether elevated VSIG4 expression reflects deleterious changes that occur with age. To evaluate the relationship between VSIG4 expression and health, we conducted a cross‐sectional study of a single cohort of male and female NIH Swiss mice where physiological frailty index (PFI), a diverse multi‐parameter assessment of animal health (Antoch et al., 2017), was measured at multiple ages. In parallel, gWAT was collected for assessing VSIG4 expression *via* immunoblot analysis. These samples revealed an age‐dependent increase in VSIG4 expression, with densitometric analysis revealing at least a ninefold increase in both male and female mice up to ages 132 and 108 weeks, respectively (Figure 4a–d). Notably, gender‐specific differences were observed in the kinetics of acquired VSIG4 expression; while male mice exhibited a consistent increase in VSIG4 expression starting at 78 weeks old, increased expression of VSIG4 in females was observed as early as 44 weeks old and plateaued at 82 weeks old. By comparison, little to no change was observed in the expression of F4/80 between aged cohorts. Significant heterogeneity exists in the expression of VSIG4 in gWAT among middle‐aged male mice. We sought to determine whether this variation was reflective of differences in health between mice, as assessed by PFI. In male and female mice, PFI showed a strong correlation with age in the subset of mice analyzed for VSIG4 expression (*r* = .82 and .70, respectively; *p* ≤ .01; Figure 4e,h). In male mice, VSIG4 expression in gWAT was strongly correlated with both age and PFI (*r* = .84 and .82, respectively; *p* ≤ .002; Figure 4f,g). In female mice, VSIG4 expression was strongly correlated with age (*r* = .80; *p* = .002) but lacked a statistically significant correlation with PFI (*r* = .42; *p* = .17; Figure 4i,j).

Aging is associated with the accumulation of a heterogeneous population of cells expressing elevated levels of CDK inhibitor protein p16*^Ink4a^*, the clearance of which, *via* genetic means or chemical intervention (e.g., senolytics), has demonstrated an improvement of health and/or extension of median life span across multiple studies (Baker et al., 2011; Xu, Palmer, et al., 2015; Xu et al., 2018; Yousefzadeh, Zhu, et al., 2018). To determine whether VSIG4 expression was associated with the presence of p16*^Ink4a^*‐positive cells in adipose tissue, gWAT was analyzed from young (18‐week‐old) and aged (96‐week‐old) female INK‐ATTAC mice, a strain that enables the detection of cells with elevated p16*^Ink4a^* promoter activity (Yousefzadeh, Zhu, et al., 2018). Mice of each age group were acutely treated with vehicle or the senolytic flavonoid fisetin (100 mg/kg for two consecutive days by oral gavage) recently shown to deplete p16*^Ink4a^*‐positive cells *in vivo* and extend murine healthspan (Yousefzadeh, Zhu, et al., 2018; Zhu et al., 2017). Analysis of the proportion of p16*^Ink4a^* cells (*via* CyTOF) and VSIG4 expression revealed age‐dependent increases among gWAT samples from vehicle‐treated mice (Figure 4k,m; Figure S9). However, within each sample, the level of p16*^Ink4a^*‐positive cells in perigonadal fat did not correlate with VSIG4 expression (Figure 4l). Further, while fisetin treatment abrogated the increase in p16*^Ink4a^*‐positive cells with age, the increase in VSIG4 was unaffected (Figure 4k,m). Expression of F4/80 was similar across groups, suggesting that Mφ numbers were unaffected by both age and treatment (Figure 4n; Figure S9). Taken together, these data indicate that the p16*^Ink4a^*‐positive cell burden en masse does not drive expression of VSIG4 in ATMs in female mice.

## 3 DISCUSSION

The pathogenesis of age‐related diseases in mice and humans is thought to be driven in large part by deleterious changes in adipose tissue over time that give rise to systemic tissue dysfunction through its continuous secretion of pro‐inflammatory cytokines. As sentinels of the immune system and mediators of inflammatory response, Mφ play a central role in maintaining tissue homeostasis. Phenotypic changes in ATMs likely reflect processes underlying and/or contributing to increases in physiological deficits with age, yet these cells remain largely uncharacterized. Here, we performed an unbiased transcriptome analysis of an ATM‐enriched population of SVF cells to elucidate age‐related differential gene expression.

Analysis of age‐associated gene upregulation of ATM‐enriched SVF revealed inflammatory cytokines and chemokines previously associated with aged ATMs as drivers of “inflammaging”: *Il6*,* Ccl2*,* Cxcl1*, and *Cxcl2* (Garg et al., 2014; Lumeng et al., 2011). However, consistent with other studies, the analysis of these inflammatory markers in total SVF did not reveal increased gene expression in aged mice (Liu, Shen, Ueno, Patel, & Kraemer, 2011; Starr et al., 2013; Wu et al., 2007). Most genes identified in our study are not exclusively expressed by Mφ, and age‐related differences in ATMs can be diluted within SVF or in whole adipose tissue by other expressing cell types. Moreover, expansion of the B‐ and T‐lymphocyte population in adipose tissues with age further reduces the proportion of Mφ, whose numbers remain largely unchanged (Bapat, Myoung Suh, et al., 2015). A notable caveat of this study is that the ATM enrichment procedure developed relies on their phagocytic ability that may enrich for a subpopulation of highly phagocytic ATMs and/or elicit phagocytosis‐related changes in gene expression (A‐Gonzalez et al., 2017). While differential age‐dependent responses of ATMs to various stimuli are likely relevant to changes in adipose with age (Starr et al., 2009), in this study we focused on identifying stable Mφ‐specific aging biomarkers that could be assessed with minimal tissue processing. Importantly, we confirmed that our strongest marker, VSIG4, was reliably upregulated in aged Mφ from intact adipose tissue for both male and female mice in two genetic backgrounds (C57BL/6J and NIH Swiss). Gender‐specific differences in expression level and temporal kinetics were observed. Female mice expressed lower levels of VSIG4 than males at a young age and exhibited increased expression earlier in life that plateaued at 80 weeks of age. In contrast, VSIG4 expression in males began to show a consistent increase starting around 80 weeks of age, with expression strongly correlated with physiological frailty index. Together, our results indicate that VSIG4 expression in adipose tissue is a novel biomarker of chronological and biological aging in mice.

As both an immune checkpoint protein and complement receptor, VSIG4 plays central roles in modulation of innate and adaptive immune responses. VSIG4, a transmembrane protein with extracellular immunoglobulin domains, is a tissue‐resident Mφ marker primarily expressed by liver Kupffer cells in mice, with low‐level expression present in the heart, lungs, spleen, thymus, and peritoneal cavity (Helmy et al., 2006; Lee et al., 2006; Pinto et al., 2012; Vogt et al., 2006). In humans, VSIG4 is highly expressed in Mφ in the liver, lungs, adrenal glands, and placenta (Helmy et al., 2006). Elevated expression of VSIG4 occurs in the context of autoimmune disease and cancer in both mice and humans [e.g., foam cell Mφ (Lee et al., 2006), synovial Mφ in arthritic joints (Lee et al., 2006), Mφ in tumor microenvironment (Liao et al., 2014; Sturtz et al., 2014; Xu, Jiang, et al., 2015)], and has been proposed as a diagnostic and prognostic indicator of several diseases, including cancer, preeclampsia, and rheumatoid arthritis (Roh et al., 2017; Textoris et al., 2013; Xu, Jiang, et al., 2015; Zheng et al., 2016). Recently, elevated levels of circulating VSIG4 have been proposed as a surrogate marker for lymphoma‐associated hemophagocytic lymphohistiocytosis, a syndrome of severe inflammation resulting from the expansion of activated Mφ and lymphocytes that are defective in cytotoxic functions (Yuan et al., 2020). Future studies are needed to determine whether VSIG4 levels in plasma reflect tissue‐related changes with age and can serve as a biomarker of healthspan and/or life span.

VSIG4, a member of the B7 family‐related immune checkpoint proteins, was shown to be a potent inhibitor of T‐lymphocyte activation, suppressing proliferation, cytokine response, and helper T cell‐mediated isotype switching in B lymphocytes (Jung, Joo, Lim, & Choi, 2015). VSIG4 signaling plays a critical role in the induction of regulatory T cells (Treg), a subset of helper T cells that aids in the establishment of peripheral immune tolerance (Yuan, Yang, Dong, Yamamura, & Fu, 2017). Further, VSIG4 does not suppress the expansion of activated Tregs unlike conventional CD4+ and CD8+ effector T cells. Tregs accumulate in visceral adipose tissue with age expressing a unique biomarker, ST2 (Bapat, Suh, et al., 2015; Cipolletta, Cohen, Spiegelman, Benoist, & Mathis, 2015). Clearance of the ST2‐positive Treg population alleviated age‐induced insulin resistance, suggesting that their accumulation plays a role in systemic metabolic dysfunction (Kolodin et al., 2015). The interplay between Mφ and T lymphocytes is highly relevant to aging and represents a potential area of therapeutic intervention, as the modulation of Mφ (*via* activation by IL‐2/agonist anti‐CD40 immunotherapy) was shown to rescue age‐related deficiencies in T lymphocytes (Jackaman, Dye, & Nelson, 2014; Jackaman et al., 2013). The potential role of VSIG4‐positive ATMs in the modulation of T‐ and B‐lymphocyte phenotypes in aged adipose tissue warrants future research.

A survey of several adipose tissue depots revealed that VSIG4 was consistently upregulated in gWAT and iWAT, but not perirenal WAT. VSIG4 was absent from brown adipose tissue and did not appear with age. Adipocytes from adipose tissue depots are highly heterogeneous, expressing unique developmental gene signatures and exhibiting many functional and metabolic differences (Schoettl, Fischer, & Ussar, 2018). These differences are thought to underpin adipose tissue depot‐specific associations with inflammation and risk of metabolic syndrome in response to various factors, such as diet and age. Li *et al*. recently demonstrated that VSIG4 suppresses visceral adipose tissue inflammation in a model of DIO, wherein VSIG4 deficiency accelerated weight gain and onset of metabolic syndrome (Li et al., 2017). However, this study did not ascertain whether VSIG4 was upregulated in wild‐type mice in response to obesity‐induced dysfunction. Our data indicate that VSIG4 expression was unchanged in both gWAT and iWAT following several months of feeding on a Western‐like high‐calorie diet. These data are consistent with findings that Mφ accumulating in the fat of obese mice do not acquire *Vsig4* expression (Lumeng et al., 2007). Thus, while the presence of VSIG4 appears to play a role in promoting adipose tissue homeostasis during the onset of diet‐induced obesity, our data suggest that age, but not diet‐induced obesity, induces a significant accumulation of VSIG4‐positive ATMs in gWAT.

Analysis of VSIG4 expression across tissues revealed that age‐related upregulation was not restricted to adipose tissues, as a strong increase was also observed in the heart and thymus. In aged mice, VSIG4‐positive Mφ were found in remnants of the thymic cortex following involution, a process of age‐related thymic atrophy, conversion to adipose tissue, and immunosenescence (Thomas, Wang, & Su, 2020). The potential role of VSIG4 in the suppression of conventional T‐lymphocyte proliferation and accumulation of Tregs associated with thymic involution warrants investigation.

VSIG4‐mediated suppression of cytotoxic T lymphocytes and inflammatory responses (*via* administration of a VSIG4‐Ig fusion protein) exerts a therapeutic benefit in experimental models of autoimmune disease (Chen, Muckersie, Luo, Forrester, & Xu, 2010; Jung et al., 2015; Katschke et al., 2007). However, VSIG4 expression within the tumor microenvironment is associated with increased tumor growth and poor prognostic outcomes for several cancers (Bianchi‐Frias et al., 2018; Byun et al., 2017; Roh et al., 2017; Xu, Jiang, et al., 2015). VSIG4 may play a direct role in cancer progression, as Vsig4 deficiency was shown to suppress the growth of Lewis lung carcinoma (LLC1) isografts (Liao et al., 2014). Here, we observed that VSIG4 expression was upregulated in lungs of aged mice bearing spontaneous tumors and in an A549 lung carcinoma xenograft model. In both cases, expression of VSIG4 was strongly induced in the adjacent normal tissue. Similarly, VSIG4 was previously identified as a marker highly upregulated by invasive breast cancer near tumor‐adjacent adipose tissue compared to a distant site from the same breast (Sturtz et al., 2014). VSIG4 is also locally upregulated within the microenvironments of inflammatory diseases, such as arthritis (Lee et al., 2006; Zheng et al., 2014). The regulation of VSIG4 expression is poorly understood. Elucidation of the factors responsible for the local induction of VSIG4 within a subset of Mφ associated with inflammatory lesions and cancer may provide insight into the mechanisms that drive VSIG4 upregulation within specific tissues with age.

In addition to suppression of T‐lymphocyte response, VSIG4 also exerts anti‐inflammatory activity through its function as a complement receptor, interacting with complement component 3 cleavage products C3b and iC3b to promote non‐inflammatory clearance of opsonized pathogens and cell debris and inhibiting alternative complement pathway activation and associated inflammation (Chen et al., 2010; Helmy et al., 2006; Katschke et al., 2007; Kim et al., 2008; Nagre et al., 2018). Recent studies have also demonstrated that VSIG4 signaling intrinsically suppresses inflammatory responses *via* (a) activation of the JAK2‐STAT3‐A20 pathway leading to the suppression of NF‐κB, NLRP3 inflammasome, and IL‐1β and (b) through PI3K‐AKT‐STAT3‐mediated metabolic reprogramming (Huang et al., 2019; Kim et al., 2013; Li et al., 2017; Nagre et al., 2018).

The accumulation of senescent cells and their bioactive secretions (i.e., SASP) is implicated as local and systemic pathogenic agents, with adipose tissue a major site of senescent cell burden with age (Stout et al., 2017). In addition, the proportion of Mφ expressing p16*^Ink4a^* and β‐galactosidase (two classical markers of senescence that may indicate Mφ activation rather than bona fide senescence) increases in adipose tissue with age and likely contributes to inflammaging and factors driving age‐related changes in adipose tissue (Hall et al., 2016, 2017; Yousefzadeh, Zhu, et al., 2018). Here, we demonstrate that the proportion of cells with activated p16*^Ink4a^* promoter expression in INK‐ATTAC mice was not associated with the level of VSIG4 detected in whole gWAT from female mice. This may reflect gender‐specific differences in aging; for NIH Swiss mice, males showed a more robust induction of VSIG4 than female mice that was strongly correlated with physiological frailty. However, p16*^Ink4a^*‐positive cells are highly heterogeneous in cell lineage and expression profile, and therefore, we cannot rule out the possibility that the presence of a subset of senescent cells and/or p16*^Ink4a^*‐positive cells that express specific factors may drive the appearance of VSIG4‐positive ATMs.

We also investigated the effects of senescent cell depletion on the regulation of VSIG4 expression in INK‐ATTAC mice. These mice harbor a p16*^Ink4a^* reporter construct that enables both the detection and small‐molecule (AP20187)‐targeted elimination of cells expressing p16*^Ink4a^*. In these mice, AP20187 can eliminate all cell types expressing p16*^Ink4a^*, including Mφ. However, the extent to which VSIG4 is expressed in p16*^Ink4a^*‐positive Mφ is not yet known. We instead chose to utilize the senotherapeutic agent fisetin in our study due to its senolytic activity against a wide range of cell types (including mesenchymal cells, endothelial cells, T lymphocytes, and NK cells) but reported inability to remove p16*^Ink4a^*‐positive Mφ (Yousefzadeh, Zhu, et al., 2018). In this way, regulation of VSIG4 expression in Mφ could be assessed without the confounding influence of Mφ depletion. In addition, fisetin directly exerts immunosuppressive effects against Mφ and other cell types that may also contribute to its therapeutic benefit (Kim, Kim, Kim, & Cho, 2015; Liu et al., 2010; Paul, Majhi, Mitra, & Ray Banerjee, 2019; Zheng, Ock, Kwon, & Suk, 2008). Acute treatment of mice with fisetin was unable to influence the short‐term expression of VSIG4, indicating that neither the senolytic nor immunomodulatory effects of fisetin were able to abruptly or substantially modulate VSIG4. Notwithstanding, VSIG4 levels may require additional time to respond to senescent cell clearance or could be impacted by other senolytics that target different senescent cell types than fisetin. Whether VSIG4 levels are a suitable pharmacodynamic marker of improved tissue homeostasis following preventive and/or interventional therapies against senescent cells, other mechanisms of aging, or inflammatory diseases is an exciting prospect that warrants investigation.

Investigation of age‐related changes in ATMs reveals an increase in several M2‐related markers associated with an anti‐inflammatory phenotype, and yet, exhibits increases in other markers associated with M1 polarization and exaggerated pro‐inflammatory responses (van Beek et al., 2019; Zeyda et al., 2007). Despite several mechanisms through which VSIG4 exerts anti‐inflammatory effects, including both intrinsic and extrinsic signaling, the extent to which VSIG4 expression exerts anti‐inflammatory effects in aged mice is unknown. For instance, recently a transmembrane protein, TRIM72, was shown to directly bind to and antagonize VSIG4 signaling, resulting in an elevated inflammatory response following bacterial clearance (Nagre et al., 2018). In addition, depending on the underlying mechanisms, VSIG4 expression can promote pathogenic inflammation and disease progression. For example, VSIG4 was found to exacerbate an experimental model of colitis in which NLRP3 inflammasome activation is associated with limiting disease progression (Huang et al., 2019).

**FIGURE 5:**
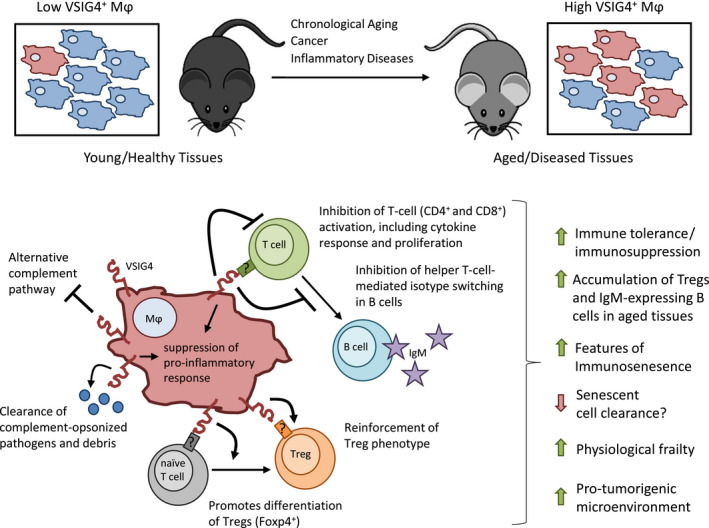
Schematic of a proposed model of VSIG4‐driven increase in physiological frailty with age. (a) Our research indicates that VSIG4 expression accumulates throughout various tissues with age, notably white adipose tissue depots. In addition, VSIG4 expression is strongly associated with site of cancer growth and several inflammatory diseases. (b) VSIG4 expression promotes immune tolerance through its immunosuppressive activities, including (i) intrinsic suppression of Mφ responses following VSIG4 activation, (ii) efficient clearance of complement‐opsonized pathogens and debris, (iii) promotion of Treg differentiation and reinforcement of Foxp3^+^ Treg phenotype, (iv) suppression of conventional CD4+ and cytotoxic CD8+ T‐lymphocyte activation, cytokine response, and proliferation, and (v) suppression of T cell‐mediated isotype switching in B lymphocytes. We demonstrated that VSIG4 expression in gWAT strongly correlates with physiological frailty in male mice. This schematic depicts scenarios in which VSIG4 activities may promote age‐related tissue dysfunction. VSIG4‐mediated suppression of conventional CD4+ and cytotoxic CD8+ T‐lymphocyte response and expansion and a shift toward CD4^+^ Foxp4^+^ Treg phenotypes may help to drive immunosenescence of T lymphocytes. Further, the suppression of both innate and adaptive immunity and promotion of immune tolerance by VSIG4 encourage a pro‐tumorigenic microenvironment and may prevent efficient clearance of damaged and/or senescent cells, resulting in unresolved inflammation and tissue dysfunction

In this study, we have identified VSIG4, a multifaceted checkpoint protein of recent interest in fields of inflammatory disease and cancer, as a novel biomarker of aging Mφ in tissues associated with age‐related systemic inflammation and immunosenescence. In male mice, VSIG4 accumulation was strongly correlated with increased physiological frailty, suggesting that this novel biomarker reflects a fundamental process underlying natural organismal aging. Based on the current understanding of VSIG4 function, we present here a hypothetical model detailing how VSIG4 upregulation may drive age‐related changes (Figure 5). However, future studies are required to elucidate the mechanism of VSIG4 upregulation and to evaluate whether VSIG4‐expressing Mφ in adipose and/or other tissues (e.g., thymus) directly contribute to life span or age‐related deficits.

## 4 EXPERIMENTAL PROCEDURES

### 4.1 Animals

Male and female C57BL/6J mice were obtained from Jackson Laboratory (Bar Harbor, ME) at 6–8 weeks of age, and Cr:NIH(S) mice (NIH Swiss) were obtained from Charles River (Wilmington, MA) at 6 weeks of age and were allowed to age at the Roswell Park Comprehensive Cancer Center (RPCCC, Buffalo, NY) animal facility. Mice housed at RPCCC were fed autoclaved standard chow (Teklad Global 18% Protein Rodent Diet) and sterile drinking water *ad libitum*. For studies with high‐calorie diet, 9‐week‐old male C57BL/6J mice were fed *ad libitum* irradiated “Western”‐type high‐fat diet (TD.88137; 42% kCal from fat) or control diet (TD.08485; 13% kCal from fat) from Harlan Teklad for up to 8 months. The fasting glucose and intraperitoneal glucose tolerance test (IPGTT) (1 g/kg of body weight) was performed after 16 hr of fasting. Blood glucose concentration was immediately measured using an AlphaTRAK 2 Blood Glucose Monitoring Kit (Abbott Laboratories) from blood drawn from a cut at the tip of the tail. Severe combined immunodeficiency (SCID) mice (C.B‐Igh‐1blcrTac‐Prkdcscid/Ros) were obtained from the RPCCC animal facility. A549 xenografts (10^6^ cells) were subcutaneously inoculated into the dorsal flank of male SCID mice. All the animals were confined to a limited access facility with environmentally controlled housing conditions throughout the entire study period and maintained at 18–26°C, 30%–70% air humidity, 12‐hour light/dark cycle. The animals were housed in micro‐isolation cages under pathogen‐free conditions. Animal procedures were performed in compliance with the guidelines approved by the Institutional Animal Care and Use Committee (IACUC) of RPCCC. At Mayo Clinic (Rochester, MN), female heterozygous INK‐ATTAC mice (a mixed 129 × C57BL/6 × FVB genetic background) were treated with fisetin (100 mg/kg delivered *via* oral gavage) for two consecutive days, and 5 days later, white adipose tissues were collected for CyTOF analysis, as previously described (Yousefzadeh, Zhu, et al., 2018). A portion of each whole adipose tissue sample was freshly frozen and stored for subsequent immunoblot analysis at RPCCC.

### 4.2 Physiological Frailty Index

The following parameters were measured for the assessment of animal health at various ages, and Physiological Frailty Index (PFI) was calculated, as previously described (Antoch et al., 2017): body mass index, grip strength, non‐invasive hemodynamic parameters (systolic, diastolic and mean blood pressure, heart rate, tail blood flow, and tail blood volume), whole blood cell counts, plasma concentration of cytokines (CXCL1, CRP) and triglycerides, and blood concentration of fasting glucose and insulin. PFI was calculated for each individual mouse as compared to a reference group of young mice (26‐week‐old male, *n* = 20; 30‐week‐old female, *n* = 19) from the same cohort. For each parameter, individual values were assigned a deficit score based on the number of standard deviations away from the mean of the young control group. PFI was calculated as the ratio of the deficit score aggregate to the total number of parameters measured.

### 4.3 Liposomal clodronate treatment

Mice were injected intraperitoneally with 200 μl of liposomal clodronate (Clodrosome^®^, 5 mg/ml) or liposomal PBS (Encapsome^®^ vehicle). At 1 week post‐injection, adipose tissues were collected and snap‐frozen in liquid nitrogen. Liposomal reagents were obtained from Encapsula NanoSciences.

### 4.4 Isolation of gWAT stromal vascular fraction

Perigonadal white adipose tissue was collected from mice into pre‐weighed conical vials containing PBS on ice. Isolation of SVF cells from gWAT for flow cytometric analysis was performed using Collagenase II (Sigma) digestion of minced adipose tissue, as described (Cho, Morris, & Lumeng, 2014). For isolation of SVF for RNA extraction, cells were isolated from minced adipose tissue utilizing the Adipose Tissue Dissociation Kit used in combination with the GentleMACS™ dissociator (Miltenyi Biotec), according to the manufacturer's instructions.

### 4.5 Macrophage enrichment of SVF

A single‐cell suspension of stromal vascular fraction (SVF) from pooled gWAT was isolated using the Adipose Tissue Dissociation Kit from Miltenyi Biotec, according to the manufacturer's instructions. Cell pellets were resuspended in tissue culture medium (DMEM supplemented with 10% heat‐inactivated FBS), and cell concentration was adjusted to 4 × 10^6^ cells/ml. Next, 1 ml of superparamagnetic nanoparticles suspension (~150‐nm EasySep™ Magnetic Particles; StemCell Technologies) was washed twice for 5 min with 2.5 ml of medium using an EasySep™ Magnet, and nanoparticles were resuspended in 1 ml of medium. The SVF cells were then mixed with nanoparticles (5 ml of cell suspension per 1 ml of pre‐washed nanoparticles) and incubated for 2 hr at 37°C with intermittent agitation. Following incubation, cells that phagocytized the magnetic particles were enriched *vi*a positive magnetic‐activated selection for 5 min using EasySep™ Magnets. Next, the selected cells were washed 3 times for 5 min each with 2.5 ml of medium on magnets, yielding the Mφ‐enriched SVF. The non‐selected cells at each selection step were pooled, yielding a Mφ‐depleted SVF. After pelleting, both fractions were resuspended in 1 ml of medium, counted, and immediately used for RNA extraction (at least 1.5 × 10^6^ cells per sample) and/or immunophenotyping.

### 4.6 RNA extraction

Total RNA was extracted from stromal vascular fraction cells using QIAzol^®^ and isolated *via* an miRNeasy Mini Kit (Qiagen). For whole adipose tissue, frozen gWAT was disrupted using a tissue homogenizer (Omni International) in the presence of QIAzol^®^, and total RNA was isolated *via* an RNeasy Lipid Tissue Mini Kit (Qiagen). Kits were utilized as per the manufacturer's instructions. RNA was quantitated *vi* *a* Qubit, and the quality was assessed *via* Agilent TapeStation 4200 for RNA‐seq and NanoString applications. The RNA integrity number (RIN) was greater than 7 for all samples analyzed.

### 4.7 RNA sequencing

The sequencing libraries were prepared from total RNA (700 ng) using the TruSeq Stranded Total RNA Kit (Illumina, Inc), following the manufacturer's instructions. Adapter‐ligated libraries were amplified by PCR, purified using AMPure XP beads, and validated for appropriate size on a 4200 TapeStation D1000 Screentape (Agilent Technologies, Inc). The DNA libraries were quantitated using a KAPA Biosystems qPCR Kit and were pooled together in an equimolar fashion. The pooled libraries were then denatured and diluted to 16 pM for On‐Board Cluster Generation and sequencing on a HiSeq 2500 sequencer using the appropriate single‐read cluster kit and rapid mode SBS reagents for 50‐cycle single‐read sequencing following the manufacturer's recommended protocol (Illumina, Inc.). The RNA sequencing data from this study have been deposited in the NCBI Gene Expression Omnibus (GEO) under the accession number GSE154832 (http://www.ncbi.nlm.nih.gov/geo/).

### 4.8 RNA‐seq analysis

Reads were trimmed using Trimmomatic v0.36 (Bolger, Lohse, & Usadel, 2014), and read quality was estimated using FastQC (Andrews, 2013). The reads were aligned to the mouse genome (mm10) with TopHat v2.1.0 (Trapnell, Pachter, & Salzberg, 2009) in very‐sensitive mode. Transcript counts were obtained using HTSeq‐count (Anders, Pyl, & Huber, 2015). Differential expression was calculated from DESeq‐normalized expression values (Anders & Huber, 2010).

### 4.9 NanoString gene expression array

Mφ‐associated genes exhibiting age‐related differential gene expression in RNA‐seq analyses were chosen for downstream validation by NanoString, a platform for direct RNA expression quantitation of multiple target genes from a single sample. For each sample, 100 ng of RNA was analyzed *via* NanoString custom codeset to measure the expression of 20 candidate genes, 5 internal reference genes (*B2 m*,* Sap130*,* Sdha*,* Tbp*,* and Tubb5*), 3 Mφ‐specific markers (*Adgre1*,* Csf1r*,* and Mrc1*), 8 external RNA control consortium (ERCC)‐negative controls, and 6 ERCC‐positive spike‐in controls, following the manufacturer's instructions (NanoString Technologies). Gene expression in pooled RNA extracted from whole fat (*n* = 3–6 mice per sample), or in RNA extracted from pooled SVF with and without Mφ enrichment (*n* = 3–10 mice per sample), was analyzed.

Raw count data derived from the NanoString platform were pre‐processed by normalizing to NanoString ERCC‐positive controls and to the 5 internal reference genes using the nSolver™ NanoString analysis software (v3.0). Genes were classified as having low or undetectable expression through comparison to the 8 ERCC‐negative control transcripts from the analyzed samples; target gene expression within 2 standard deviations of the negative control mean was considered undetectable and given this threshold value (value = 42) for subsequent calculations. Expression values were adjusted to account for differences in either internal reference genes or Mφ‐specific genes between samples by normalizing against the average of relative expression values for each reference gene across all samples analyzed. Error bars were calculated by propagating error associated with the reference gene normalization.

### 4.10 Flow cytometry

Peritoneal cells were harvested by lavage using 8 ml of cold saline containing 2% heat‐inactivated FBS. A single‐cell suspension of gWAT stromal vascular fraction was obtained *via* Collagenase II digestion of minced tissue. After blocking Fc receptors (anti‐CD16/CD32 antibodies, clone 93; eBioscience) for 10 min, live cells were stained in Cell Staining Buffer (BioLegend) with the following fluorochrome‐conjugated antibodies against cell surface receptors: FITC‐labeled anti‐CD45.2 (104; eBioscience), V500‐labeled anti‐CD11b (M1/70, BD Horizon), APC‐eFluor 780‐labeled anti‐F4/80 (BMB; eBioscience), and APC‐labeled anti‐VSIG4 (NLA14; eBioscience). After incubation for 30 min on ice in the dark, cells were washed and resuspended with Cell Staining Buffer. To distinguish dead cells, cell membrane‐impermeant DNA stain BOBO™‐3 (Molecular Probes) was added to the cell suspension (20 nM final concentration) 3 min prior to acquisition. Data acquisition for 5 × 10^5^ to 1 × 10^6^ cells was performed on a custom BD LSR™ II instrument at the Roswell Park Cancer Institute FACS facility using BD FACSDiva™ Software (BD Biosciences). Data compensation and analysis were performed using FCS Express 4 (De Novo Software). Compensation was performed using data from single‐color antibody staining controls prepared with either OneComp beads (eBioscience) or, in the case of viability staining, single‐stained cell suspensions. Cell populations were identified *via* a sequential gating strategy (see Figure S6A,B). Gating was delineated utilizing “Fluorescence‐minus‐one” (FMO) controls for all antibodies, where a fluorophore‐labeled antibody of interest was substituted in the full‐color staining cocktail with the corresponding isotype control antibody conjugated with the same fluorophore: FITC‐labeled mouse IgG2a κ (eBM2a; eBioscience), V500‐labeled rat IgG2b κ (A95‐1; eBioscience), APC‐eFluor 780‐labeled rat IgG2b κ (eB149/10H5; eBioscience), and APC‐labeled rat IgG2a κ (eBR2a; eBioscience). FMO controls were established separately for cells from both young and old mice to account for potential differences in autofluorescence. The intensity of APC‐conjugated anti‐VSIG4 staining is presented as the median fluorescent intensity (MFI) of gated VSIG4‐positive cells minus the MFI from VSIG4‐negative cells.

### 4.11 Immunoblotting

Whole adipose tissue protein extracts were obtained *via* homogenization in RIPA lysis buffer (Sigma) supplemented with Halt phosphatase (Pierce) and protease inhibitors (Sigma). Protein concentration was assessed *via* Pierce BCA assay kit. Immunoblots were performed as previously described (Frescas et al., 2017). Briefly, protein extracts (15–30 μg) diluted into reducing sample buffer were fractionated *via* SDS‐PAGE (4%–12% Mini‐Protean TGX gel) and transferred onto a 0.45‐μm nitrocellulose membrane (all reagents obtained from Bio‐Rad;). Membranes were probed with primary antibodies against mouse VSIG4 (1:500 to 1:2000 dilution; R&D Systems) and F4/80 (1:1000 to 1:4000 dilution; GeneTex), and subsequently incubated with horseradish peroxidase (HRP)‐conjugated secondary antibodies. Alternatively, HRP‐conjugated primary antibodies against GAPDH (1:5000 dilution; clone 71.1; Sigma) or β‐actin (1:20,000 dilution; clone AC‐15; Sigma) were used for probing protein loading controls. Blots were washed thoroughly with Tris‐buffered saline containing 0.1% Tween‐20 prior to incubation with SuperSignal West Dura chemiluminescent peroxidase substrate (Thermo Scientific) and were exposed using FluorChem E System (Protein Simple). Band densitometry analysis of immunoblots was performed with ImageJ software (NIH).

### 4.12 Immunofluorescent staining

Immunofluorescence staining of whole perigonadal adipose tissue was performed as previously described (Hall et al., 2017). Briefly, adipose tissues were fixed 4 hr in 4% paraformaldehyde in PBS at 4°C, washed in PBS overnight, incubated with blocking solution (PBS with 5% normal rat serum and 0.25% Triton X‐100) 1 hr at room temperature, and stained with a cocktail of rat monoclonal antibodies against mouse F4/80 (Alexa Fluor 647‐conjugated, clone BM8, BioLegend, 1:100 dilution), mouse CD301a (Alexa Fluor 647‐conjugated, clone LOM‐8.7, BioLegend, 1:100 dilution), or mouse CD206 (Alexa Fluor 488‐conjugated, clone C068C2, BioLegend, 1:100 dilution), and goat polyclonal against mouse VSIG4 (R&D System, 1:400 dilution). Samples were stained at room temperature with mild agitation overnight, washed in 5 changes of PBS 6–8 hr at room temperature, and stained overnight with secondary donkey anti‐goat antibody conjugated with Cy3 (Jackson ImmunoResearch, 1:1000 dilution in blocking solution containing 5% normal donkey serum, PBS, and 0.25% Triton X‐100). Nuclei were counterstained with DAPI. Samples were washed 4 hr with several changes of PBS, cleared, and mounted in glycerol.

For histology and immunohistochemistry on normal tissues and xenograft tumors, samples were fresh‐frozen in Neg‐50 freezing medium (Thermo Fisher Scientific). Next, 12‐μm sections were prepared on a cryotome CM1900 (Leica Biosystems). Sections were fixed with 4% formaldehyde in PBS for 5 min at room temperature and washed three times with PBS. Sections were incubated with blocking solution 15 min at room temperature and stained for 30 min at room temperature with antibodies against Mφ markers (anti‐CD206, anti‐CD301a, and anti‐F4/80 at 1:50 dilution, anti‐VSIG4 at 1:200 dilution), anti‐α‐smooth muscle actin (Cy3‐conjugated, mouse monoclonal antibody, clone 1A4, Sigma, 1:500 dilution), anti‐collagen III (rabbit polyclonal antibody, Abcam, 1:200 dilution), anti‐CD3 (rabbit monoclonal antibody, clone SP7, Abcam, 1:100 dilution), anti‐CD68 (Alexa Fluor 488‐conjugated, rat monoclonal antibody, clone FA‐11, BioLegend, 1:50 dilution), and anti‐CK8 (rat monoclonal antibody, clone Troma I, Developmental Studies Hybridoma Bank, 1:200 dilution). For sections stained with non‐conjugated primary antibodies, sections were washed and stained with either cross‐absorbed donkey anti‐rabbit IgG (Alexa Fluor 488‐conjugated), anti‐goat IgG (Alexa Fluor 546‐conjugated), or anti‐rat IgG (Alexa Fluor 488‐conjugated) at a 1:500 dilution (Jackson ImmunoResearch), respectively. After washing with PBS, sections were mounted with ProLong Diamond antifade reagent with DAPI. For morphological analysis, unfixed sections were stained by May–Grünvald method.

All images were acquired with AxioImager Z1 (Carl Zeiss, Inc.) microscope using an AxioVision software (Zeiss). For quantitation of the proportion of VSIG4‐positive Mφ (CD206^+^), at least 100 CD206^+^ cells per sample were counted across several representative fields.

### 4.13 Statistical analyses

Unless otherwise noted, data were analyzed by unpaired Student's two‐tailed t test and are expressed as mean ± standard deviation. All statistical analyses were performed using GraphPad Prism 5.

## CONFLICT OF INTEREST

The authors have declared no conflict of interests.

## AUTHOR CONTRIBUTIONS

BMH, OBC, AVG, MPA, PAK, TT, and JLK conceived and designed the experiments. BMH, ASG, ES, PAK, DF, SV, OVL, MPA, and YZ performed the experiments. BMH, ES, PAK, and VK analyzed the data. YZ, TT, JLK, and IEK contributed reagents/materials/analysis tools. BMH wrote the manuscript. AVG, OBC, MPA, TT, and JLK edited the manuscript.

## Data Availability

The data supporting the findings of this study are openly available in the NCBI GEO database (reference number GSE154832) and in Dryad at https://doi.org/10.5061/dryad.hdr7s​qvfp (Hall et al., 2020)
