# Supplementary material for: Immune checkpoint protein VSIG4 as a biomarker of aging in murine adipose tissue
Source: Aging Cell. 2020 Aug 28;19(10):e13219. doi: 10.1111/acel.13219 (PMC7576241; doi:10.1111/acel.13219)
Supplement: Supplementary file 1 [file ACEL-19-e13219-s001.pdf]

Immune Checkpoint Protein VSIG4 as a Biomarker of Aging in Murine Adipose Tissue

Brandon M. Hall, Anatoli S. Gleiberman, Evguenia Strom, Peter Krasnov, David A. Frescas, Slavoljub Vujcic, Olga V. Leontieva, Marina P. Antoch, Valeria Kogan, Igor E. Koman, Yi Zhu, Tamara Tchkonia, James L. Kirkland, Olga B. Chernova and Andrei V. Gudkov

SUPPLEMENTAL MATERIAL

Table S1. Differential gene expression of pan-Mφ markers and internal reference genes from RNAseq analysis. Values presented as fold change.

Gene Symbol	Differential Expression: Mϕ-enriched vs Mϕ-depleted Old SVF	Differential Expression: Young vs Old Mϕ-enriched SVF
Mϕ-associated genes		
Adgre1 (F4/80; Emr1) [a]	2.0	0.9
Cd163	7.9	2.4
Cd68	3.8	0.8
Clec10a (Cd301a)	7.7	2.0
Csf1r [a]	3.4	1.1
Itgam (Cd11b)	1.1	0.8
Itgax (Cd11c)	1.4	0.5
Mrc1 (Cd206) [a]	7.5	1.6
Internal reference genes		
B2m [a]	0.8	1.0
Sap130 [a]	0.9	0.9
Sdha [a]	0.9	0.9
Tbp [a]	0.9	1.2
Tubb5 [a]	0.9	0.9

[a]Genes selected as controls for Nanostring custom code set.

Table S2. Confirmation of differential gene expression of candidates age-related genes using samples submitted for RNAseq, as analyzed by Nanostring platform. Values presented are fold change relative to young mice. nd, not determined.

Official Gene Symbol	Gene Name	Old versus Young Mϕ-enriched SVF
Candidate Genes		
Cxcl13	Chemokine (C-X-C motif) ligand 13	8.9
Cxcl5	Chemokine (C-X-C motif) ligand 5	8.5
Fcna	Ficolin A	8.1
Vsig4	V-set and immunoglobulin domain-containing 4	≥ 8.0
Saa3	Serum amyloid A 3	4.9
Ccl8	Chemokine (C-C motif) ligand 8	4.5
Lif	Leukemia inhibitory factor	4.0
Cd209g	CD209g antigen	3.8
Cd209f	CD209f antigen	3.7
Cd209d	CD209d antigen	3.4
Ccl2	Chemokine (C-C motif) ligand 2	3.3
Cxcl1 (KC)	Chemokine (C-X-C motif) ligand 1	3.1
Gpr176	G protein-coupled receptor 176	3.1
Cxcl2 (MIP2)	Chemokine (C-X-C motif) ligand 2	3.1
Il6	Interleukin 6	3.0
Inhba	Inhibin beta-A	3.0
Marco	Macrophage receptor with collagenous structure	3.0
Tacr3	Tachykinin receptor 3	≥ 1.7
Csmd1	CUB and Sushi multiple domains 1	≥ 1.4
Tmem132e	Transmembrane protein 132E	≥ 1.1
C6	Complement component 6	nd
H2-M9	Histocompatibility 2, M region locus 9	nd
Gm16548	Predicted gene 16548	nd
Mϕ-associated genes		
Adgre1 (F4/80)	Adhesion G protein-coupled receptor E1	1.0
Csf1r	Colony stimulating factor 1 receptor	1.1
Mrc1 (CD206)	Mannose receptor, C type 1	1.5
Internal reference genes		
B2m	Beta-2 microglobulin	1.1
Sap130	Sin3A associated protein	1.0
Sdha	Succinate dehydrogenase complex, subunit A, flavoprotein	0.9
Tbp	TATA box binding protein	1.2
Tubb5	Tubulin, beta 5 class I	0.9

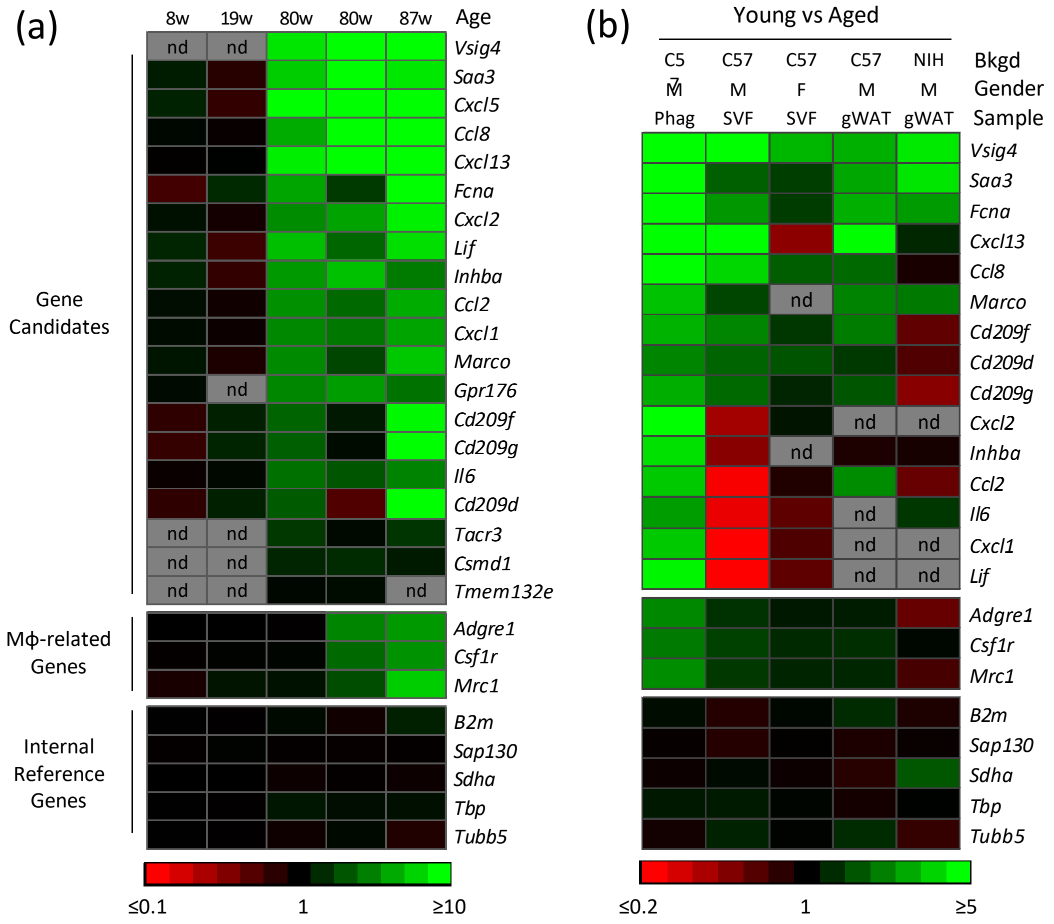

Figure S1. Nanostring analysis of 20 putative age-related genes upregulated in gWAT ATMs with age as identified by RNA-seq analysis. (a,b) Heat maps depicting fold change between pooled samples from young versus aged mice (n = 3-8 mice per pooled sample) analyzed via NanoString platform. (a) Fold differences among independent pooled samples of ATM-enriched SVF relative to the mean signal from young mice. Genes are ranked by the geometric mean of differential gene expression in aged samples. Vsig4 expression corresponds to data presented in Figure 2a. (b) Fold differences among single pooled samples of total SVF or intact gWAT from young versus aged mice. The geometric mean of ATM-enriched SVF from aged mice from (a) is depicted for comparison. Genes are ranked by the geometric mean of differential gene expression within each row, a composite value of all sample types. Gpr176 and Cxcl5 were undetectable across all samples and were excluded. Vsig4 expression corresponds to data presented in Figure 2b-e. nd, not detected.

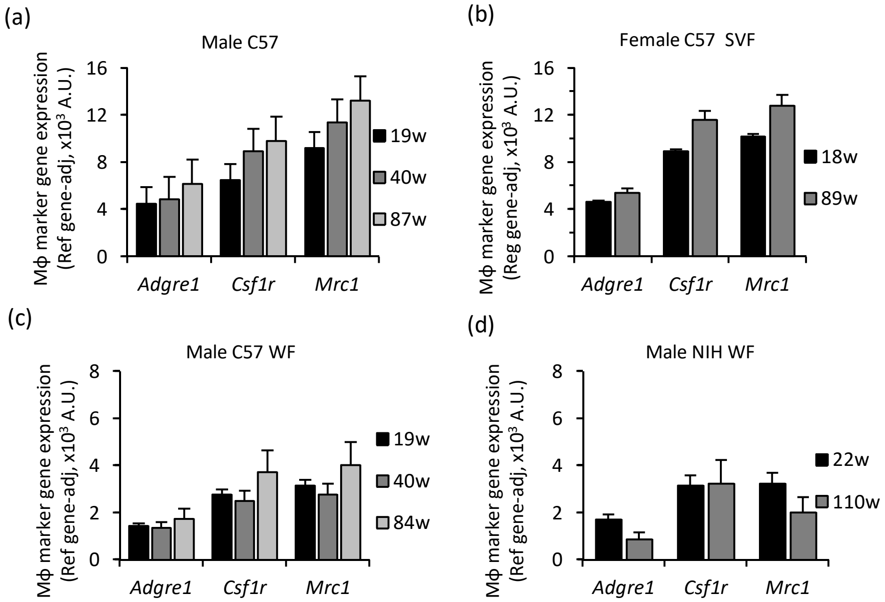

Figure S2. Age-related expression of Mϕ-associated genes in gWAT. (a-d) Nanostring gene expression analysis of Mϕ markers Adgre1, Mrc1, and Csf1r in gWAT samples from young and old mice, including analysis of total SVF of gWAT from different aged cohorts of male (a) or female (b) C57BL/6J mice, and of intact gWAT from male C57BL/6J (c) and NIH-Swiss (d) mice, adjusted to gene expression of five internal reference genes B2m, Sap130, Sdha, Tbp, and Tubb5. Data presented as a single measurement of gene expression of pooled samples (n = 3-8 mice/group) ± error propagation from SD of normalized internal reference genes. Nanostring analysis of Mϕ marker expression corresponding to Vsig4 expression presented in Figure 2b-e.

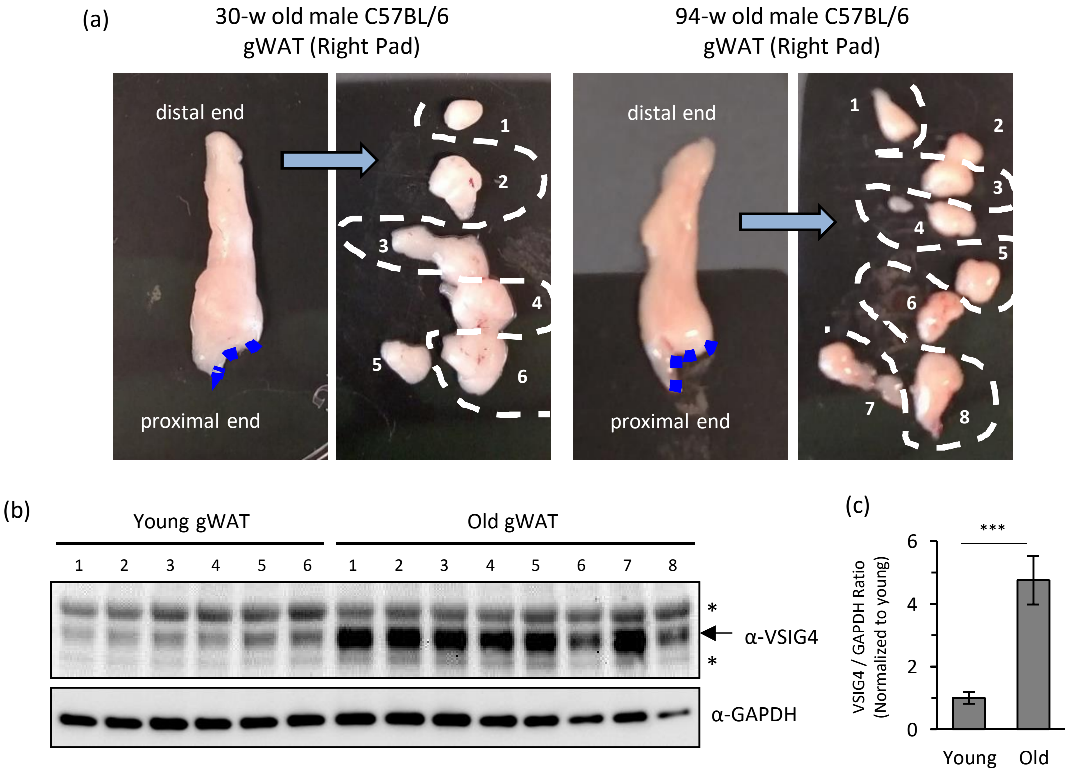

Figure S3. VSIG4 is expressed uniformly throughout young and old gWAT fat pads. (a-c) Representative experiment depicting immunoblot analysis of VSIG4 expression from a single fat pad from young (30-week old) and old (94-week old) C57BL/6J male mice. (a) The right gWAT fat pads was excised (detached from epididymis at blue dotted line) and partitioned into several pieces. (b) Anti-VSIG4 immunoblot against lysates from individual adipose tissue pieces. GAPDH was used as a loading control. (c) Denositometric analysis of the ratio of VSIG4 to GAPDH band intensity for each piece of fat was quantitated from the image in panel B. Data presented as mean ± SD, normalized to young mouse. The percent coefficient of variance among pieces of adipose tissue from a single fat pad was 27.1% and 31.4% for young and old mice, respectively. *, non-specific αVSIG4 immunoreactive bands.

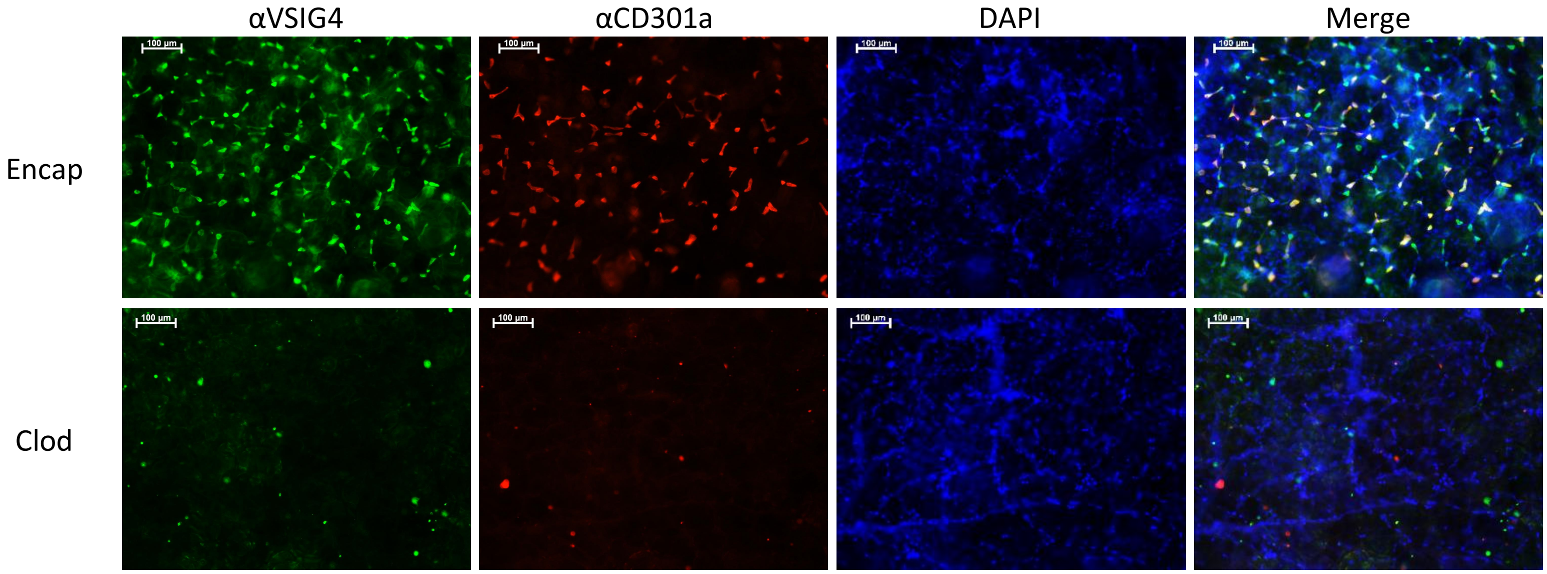

Figure S4. VSIG4 depletion in gWAT following treatment with liposomal clodronate. Representative fluorescence microphotographs of intact gWAT from old (102-week old) male C57BL/6J mice collected one week after a single administration of Encapsome (Encap; liposomal PBS) or Mϕ-depletion reagent Clodrosome (Clod; liposomal clodronate), depicting immunofluorescent staining of Mϕ-related markers VSIG4 (green) and CD301a (red), and a merged overlay with DAPI nuclear counterstain (blue). Scale bar = 100-µm. Analysis of samples corresponding to Figure 2g.

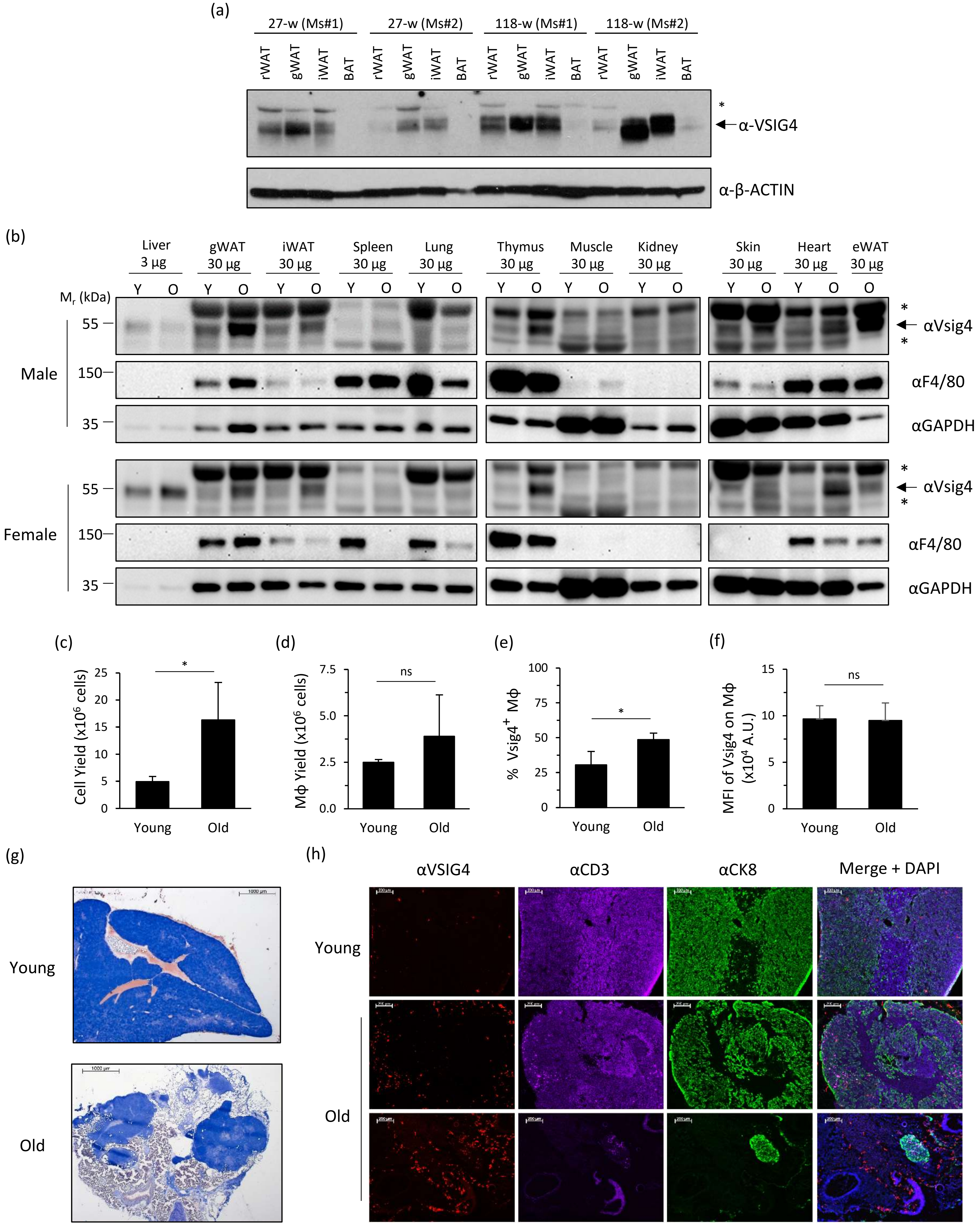

(a)
27-w (Ms#1)
27-w (Ms#2)
118-w (Ms#1)
118-w (Ms#2)
rWAT gWAT iWAT BAT
α-VSIG4
α-β-ACTIN
(b)
Liver 3 µg
gWAT 30 µg
iWAT 30 µg
Spleen 30 µg
Lung 30 µg
Thymus 30 µg
Muscle 30 µg
Kidney 30 µg
Skin 30 µg
Heart 30 µg
eWAT 30 µg
Y O
Mr (kDa)
55
150
35
Male
Female
αVsig4
αF4/80
αGAPDH
(c)
Cell Yield (x10^6 cells)
Young
Old
(d)
Mϕ Yield (x10^6 cells)
ns
(e)
% Vsig4+ Mϕ
(f)
MFI of Vsig4 on Mϕ (x10^4 A.U.)
(g)
Young
Old
(h)
αVSIG4
αCD3
αCK8
Merge + DAPI

Figure S5. Differential age-related expression changes in VSIG4 among tissues. (a) Representative immunoblot analysis of VSIG4 expression in several adipose tissue depots from individual young and old male NIH Swiss mice, including perirenal white adipose tissue (rWAT), gWAT, iWAT, and interscapular brown adipose tissue (BAT). -ACTIN was used as immunoblot loading control. *, non-specific -VSIG4 immunoreactive bands. (b) Immunoblot analysis of several tissues from young (Y; 8- to 18-weeks old) and old (O; 90- to 96-weeks old) C57BL/6J mice of both genders, where equal amounts of protein from 3 mice from each age group were pooled. Amount of protein lysate loaded is indicated (µg). (c-f) Representative experiment of flow cytometric analysis of single-cell suspensions of peritoneal lavage isolated from three individual young (19-week old) and old (120-week old) male C57BL/6J mice. All gates were defined using age-matched fluorescence minus one (FMO) controls (see gating scheme in Figure S6A,B). Quantitation of (c) total yield of viable CD45+ cells, (d) total yield of viable peritoneal macrophages (CD45+ CD11b+ F4/80+), (e) the proportion of VSIG4+ Mϕ, and (f) the median fluorescent intensity (MFI) of αVSIG4-APC stained Mϕ minus MFI from FMO control staining. Data presented as mean ± SD (n = 3 mice/group). ns, not significant ($p > 0.05$), * $p < 0.05$, compared to young mice. (g,h) Representative microphotographs of cryosections of thymus from young and aged mice, (g) stained with May-Grünwald-Giemsa for histology depicting thymic involution in aged mice (scale bar = 1,000-µm), and (h) immunofluorescent staining of VSIG4 (red), T lymphocyte marker CD3 (purple), stromal epithelial marker Collagen III (green), epithelial marker cytokeratin 8 (CK8), DAPI nuclear counterstain (blue), and merged overlay of colored channels. Images from young mice depicting healthy thymus (top row) and from old mice depicting relatively intact thymus tissue (middle row) and a region of the thymus that underwent extensive involution (bottom row). Scale bar = 200 µm.

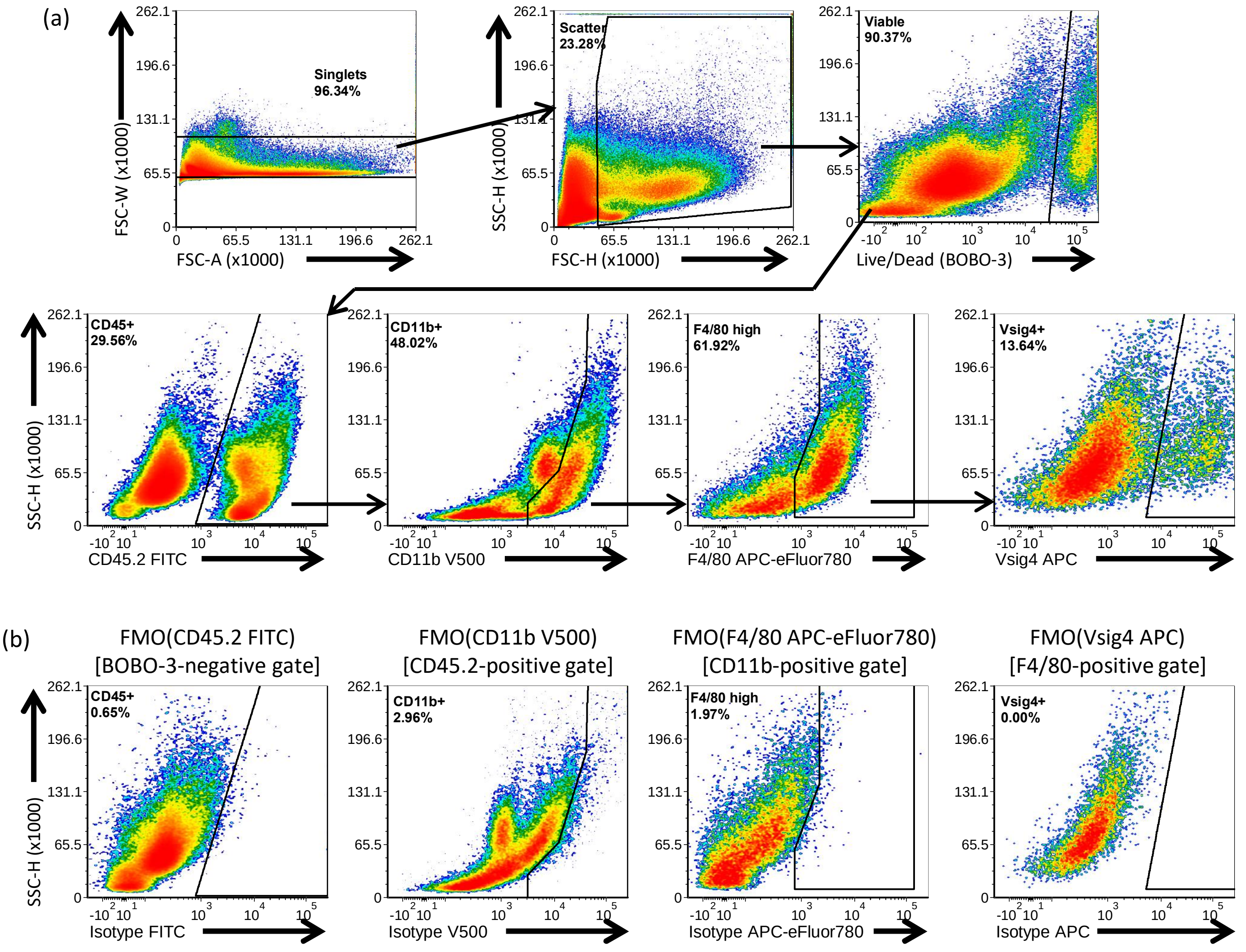

Figure S6. Gating scheme for flow cytometric analysis of VSIG4 expression in Mϕ. (a,b) Single-cell suspensions of gWAT-derived SVF from young and old C57BL/6J male mice were live-stained with immunofluorescent antibodies and BOBO-3 live/dead dye (excluded from live cells) and analyzed via flow cytometry (see Experimental Procedures). (a) Representative analysis of an individual SVF cells isolated from a young mouse. After exclusion of doublets, debris, and non-viable cells (BOBO-3+ cells) (top 3 panels, respectively), adipose-tissue Mϕ were identified (CD45.2+ CD11b+ F4/80+) for assessing the VSIG4 expression. (b) All gates in panel A utilizing fluorescent antibodies (bottom panels) were defined using age-matched fluorescence minus one (FMO) staining controls, where the indicated fluorescent antibody in the full antibody staining cocktail was replaced with an isotype control conjugated with the fluorochrome.

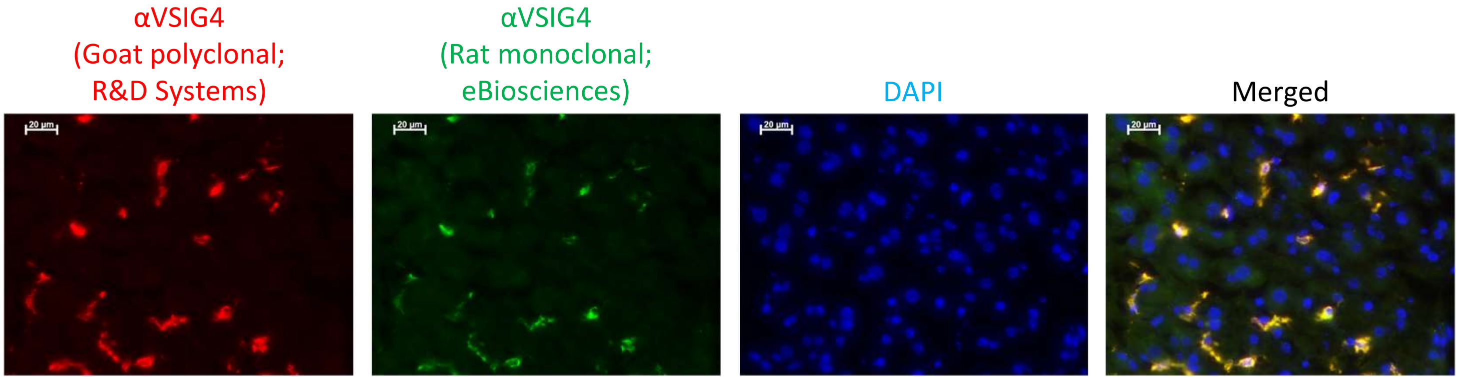

Figure S7. VSIG4 antibodies used in different applications show complete co-localization of signal in stained tissue. Representative fluorescence microphotographs of normal liver from young C57BL/6J mice depicting immunofluorescent staining of two independently-derived αVSIG4 antibodies used in this manuscript [R&D antibody used for immunoblot and tissue staining (red) and eBiosciences antibody used for flow cytometry (green)] and a merged overlay with DAPI nuclear counterstain (blue). Scale bar = 20-µm.

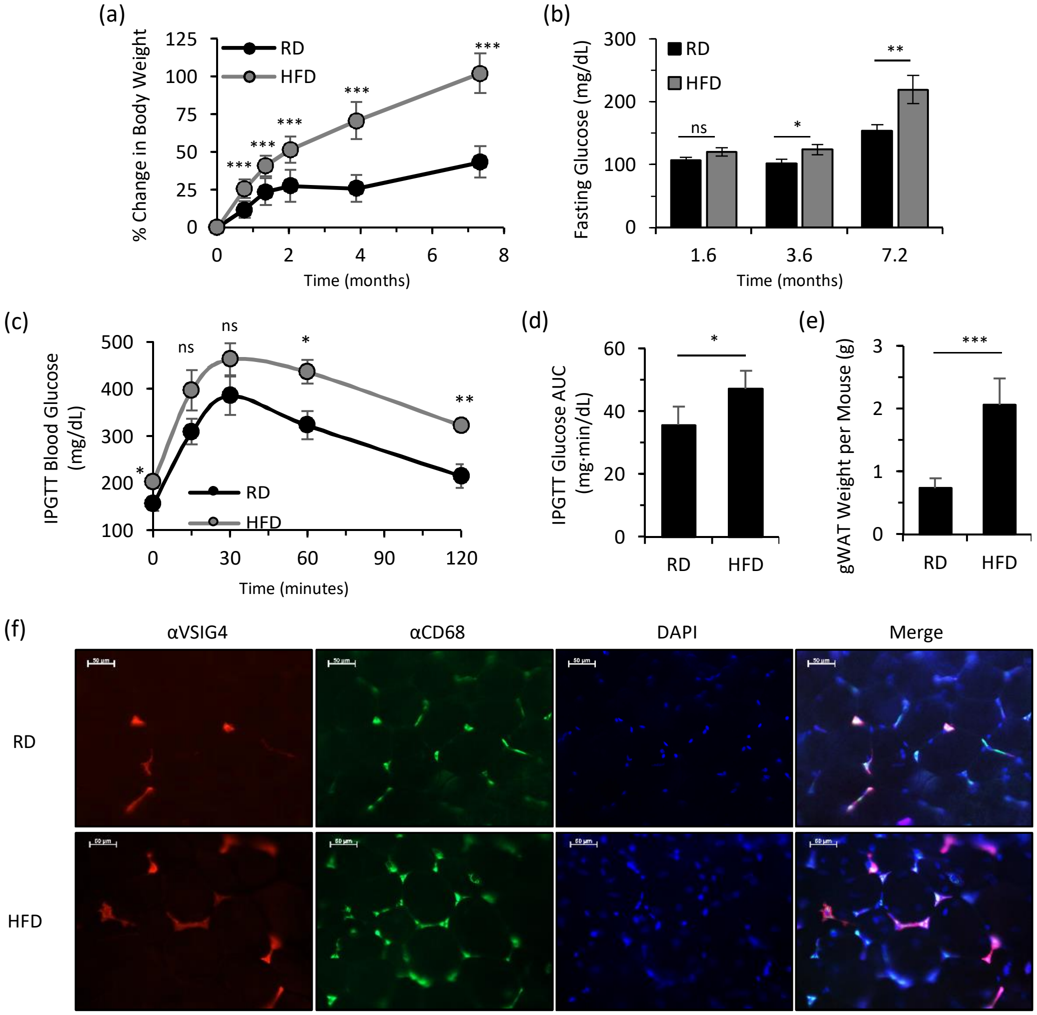

Figure S8. VSIG4 expression in a model of high-calorie diet-induced obesity. (a-f) Two-month old C57BL/6J male mice were fed a high-calorie diet ad libitum for up to 8 months. (a) Change in body weight in mice fed either regular diet (RD) or high-fat "Western" diet (HFD), relative to starting body weights (24.6 ± 0.5 grams). Data presented as mean ± SD (n = 10 mice/group). (b) Blood glucose following overnight (16-hour) fast was measured at various time points throughout high-calorie diet feeding. Data presented as mean ± s.e.m. (n = 10 mice/group). Intraperitoneal glucose tolerance test (IPGTT) was performed on RD- or HFD-fed mice and (c) blood glucose was measured over time, and (d) the area under the curve (AUC0-120min) of glucose concentration was calculated. Data presented as mean ± s.e.m. (n = 8 mice/group). (e) Weight of gWAT fad pads collected from RD- and HFD-fed mice (n = 5 mice/group). Data presented as mean ± SD. (f) Representative fluorescence microphotographs of intact gWAT from mice after 5 months of feeding RD or HFD, depicting immunofluorescent staining of Mϕ-associated markers VSIG4 (red) and CD68 (green), and a merged overlay with DAPI nuclear counterstain (blue). Scale bar = 50-µm. ns, not significant ($p>0.05$), * $p<0.05$, **$p<0.01$, ***$p<0.001$, compared to regular diet control mice at indicated time points.

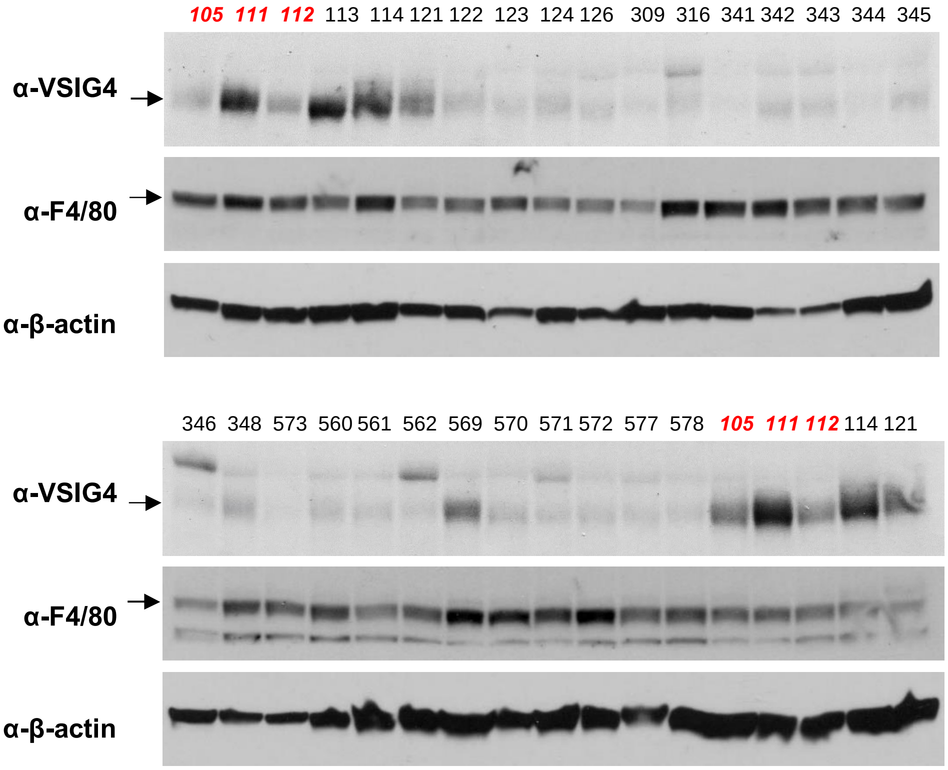

Mouse IDs			
4-month old		26-month old	
Vehicle	Fisetin	Vehicle	Fisetin
#560<br>#561<br>#562<br>#572<br>#573	#569<br>#570<br>#571<br>#577<br>#578	#105<br>#112<br>#113<br>#122<br>#123<br>#125<br>#309<br>#341<br>#343<br>#348	#111<br>#114<br>#121<br>#124<br>#126<br>#316<br>#342<br>#344<br>#345<br>#346

Figure S9. Effects of senolytic fisetin treatment on VSIG4 expression in gWAT. Analysis of gWAT samples from female INK-ATTAC mice collected 5 days after two consecutive treatments with either vehicle or fisetin (100 mg/kg per os). Representative VSIG4, F4/80, and β-ACTIN immunoblot analysis, performed at RPCCC on blinded samples provided by the Mayo Clinic (left panel). Group assignments of samples indicated (right panel). Sample IDs marked in red indicate the samples that were repeated across immunoblots and used as a standard to normalize the densitometric analysis across blots for each marker. Raw immunoblot images corresponding to data analysis presented in Figure 4k-n.
